# Stable-isotope tracing reveals the role of corticosteroid receptors in driving cortisol-mediated central and peripheral glucose regulation in zebrafish

**DOI:** 10.3389/fendo.2025.1670637

**Published:** 2025-10-14

**Authors:** Femilarani Antomagesh, Mathilakath M. Vijayan

**Affiliations:** Department of Biological Sciences, University of Calgary, Calgary, AB, Canada

**Keywords:** metabolomics, glucocorticoid receptor, mineralocorticoid receptor, intermediary metabolism, brain metabolism, stress response

## Abstract

**Rationale:**

Corticosteroids play a crucial role in the stress-induced metabolic adjustments, and this stress response is conserved across vertebrates. In teleosts, cortisol is the principal glucocorticoid and regulates metabolic processes predominantly through the activation of the glucocorticoid receptor (GR). In zebrafish (*Danio rerio*), we recently showed that both the GR and the mineralocorticoid receptor (MR) are essential for stressor perception and metabolic regulation, especially related to glucose production and target-tissue glucose uptake. Here, we tested the hypothesis that GR and MR have distinct roles in modulating the tissue-specific glucose metabolism in response to cortisol stimulation during stress in fish.

**Methods:**

This was tested using GR knockout (*nr3c1^−/−^*) and either wild-type or MR knockout (*nr3c2^−/−^*) zebrafish treated with cortisol to mimic a chronic stress condition. Stable isotope-labeled glucose (U-^13^C-glucose) was injected intraperitoneally, and the labeled intermediates were assessed to investigate the fate of the glucose carbon in the serum, liver, and brain. The metabolites in these tissues were analyzed using LC-MS to investigate the ^13^C incorporation across the metabolic pathway at a systems level.

**Results:**

Chronic cortisol stimulation enhanced glucose breakdown and its utilization in the TCA cycle for energy production. The GR and MR activation led to distinct and complementary effects on glucose utilization and the generation of TCA intermediates in the brain and liver, suggesting a tissue-specific role for these receptors in energy substrate partitioning during stress in fish.

**Conclusion:**

Overall, our results underscore the roles of GR and MR activation in elevating circulating energy substrates and facilitating tissue-level oxidative capacity and biomolecule synthesis from glucose metabolism in response to chronic cortisol stimulation in fish.

## 1 Introduction

The hypothalamus–pituitary–adrenal (HPA) axis activation in response to a stressor perception and the release of glucocorticoids (GCs) is a key adaptive response that allows animals to reestablish homeostasis (1, 2). The HPA axis activation commences with the release of the corticotropin-releasing hormone (CRH) from the hypothalamus, which, in turn, stimulates the pituitary gland to release the adrenocorticotropic hormone (ACTH), a post-translational peptide cleaved from proopiomelanocortin (1, 2). ACTH is released into the circulation and binds to the melanocortin 2 receptor (MC2R) on the steroidogenic cells of the adrenal cortex in mammals and the interrenal tissue in teleosts (HPI axis) to initiate the biosynthesis and release of GCs (2–4).

Cortisol, the principal GC in teleosts, affects various physiological functions, including metabolism, behavioral changes, ion and mineral balance, and immune functions (3–5). At the cellular level, cortisol acts through either a high-affinity mineralocorticoid receptor (MR) or a low-affinity glucocorticoid receptor (GR) (6, 7). Since stress is energy demanding, one of the predominant functions of GC is promoting energy allocation and utilization (4, 8). A well-characterized cortisol-mediated metabolic response is the increase in the circulating levels of glucose, amino acids, and fatty acid to fuel the increased energy demand associated with stress coping (4, 9).

To facilitate the increased systemic metabolite availability, cortisol exerts tissue-specific metabolic adjustments, including but not limited to promoting liver gluconeogenesis, skeletal muscle proteolysis, and inhibition of skeletal muscle glucose uptake in teleosts (3, 8, 10, 11). These responses are GR mediated due to elevated cortisol levels (12), as the high-affinity MR is activated at resting levels of cortisol (13–16). However, emerging studies using zebrafish (*Danio rerio*) lacking MR suggests a key role for this receptor in metabolic regulation during stress (6, 7). For instance, GR has been known to play an important role in stress-mediated glucose regulation (4, 11). Recently, we also showed that zebrafish lacking GR had higher glucose uptake in the muscle (7), while those lacking MR showed a lower glucose uptake and increased utilization (6). This led to the proposal that both the corticosteroid receptors are key to contributing to glucose regulation and the energy substrate management/sustenance during chronic stress in fish, but this has yet to be empirically determined.

To address this knowledge gap, we tested the hypothesis that both GR and MR activation favor a higher aerobic metabolic phenotype in the central and peripheral tissues. Specifically, the utilization of glucose in the brain and liver was assessed in response to chronic cortisol stimulation. To distinguish the contribution of central and peripheral GR and MR in glucose regulation, we treated wild-type (WT) and *nr3c2^−/−^* zebrafish (MRKO) with cortisol to mimic a chronic stress scenario, while the *nr3c1^−/−^* (GRKO) zebrafish are naturally hypercortisolemic (7). By using U-^13^C-glucose and determining the labeled and endogenous metabolites by LC/MS and isotope tracing, we were able to distinguish the contribution of GR or MR alone, or in combination, in modulating glucose flux in the brain and liver of zebrafish. Our results indicate an enhanced capacity for glucose utilization into the TCA cycle, including the generation of intermediary metabolites, in the brain and liver associated with either GR or MR activation. This study highlights the distinct and complementary role for GR and MR in facilitating tissue-specific glucose metabolism during chronic cortisol stimulation in zebrafish.

## 2 Methods

### 2.1 Zebrafish care

Adult zebrafish [Tupfel long fin strain (TL)] were maintained in a recirculating system (Tecniplast, Italy). The fish were maintained in a 14-h light:10-h dark cycle, and the water temperature, pH, and conductivity were maintained at 28.5°C, 7.5, and ~770 μS, respectively. The fish were fed with Gemma micro 500 (Skretting, USA) in the morning and live *Artemia* (San Francisco Bay Brand, USA) in the evening 5 days a week, and once with Gemma during weekends. All animal care protocols were approved by the animal care committee at the University of Calgary, and followed the guidelines set by the Canadian Council of Animal Care. Ubiquitous *nr3c1−/−* (GRKO) and *nr3c2−/−* (MRKO) zebrafish were developed using the CRISPR/Cas9 technique with a net 7-bp deletion in exon 2 of the *nr3c1* gene and a net 8-bp insertion in exon 2 of the *nr3c2* gene, respectively, as described previously (17).

### 2.2 Cortisol treatment

Age-matched 10-month-old WT male zebrafish were exposed to either a vehicle (0.05% ethanol) or cortisol (hydrocortisone; Sigma; 5 μg/mL) for 16 h (overnight), by adding to the water to elevate the whole-body cortisol levels. This treatment concentration has been shown previously to consistently and effectively elevate whole-body cortisol levels to mimic a chronic stress state (10, 18). The MRKO zebrafish were also exposed to cortisol treatment, while the GRKOs are naturally hypercortisolemic (7) and were subjected to vehicle treatment.

### 2.3 U-^13^C-glucose administration and metabolomic analysis

The fish were last fed 16 h prior to the intraperitoneal injection with 0.5 mg/g body weight of U-^13^C-labeled glucose (Sigma-Aldrich, CA, Cat: 389374). The fish were sampled at 1 h post-injection, as this time point is sufficient for entry of isotope-labeled carbon into the core metabolic pathways prior to reaching isotopic steady state, thereby reflecting differences in metabolic flow rate, as described previously (19). Blood was collected in an Eppendorf tube from caudal fin ablation and allowed to clot in ice for 10 min as described previously (19). The serum was collected from the cells by centrifugation at 1,000×*g* for 5 min for metabolomics analysis (19). The blood glucose was measured using a freestyle glucose meter and strips (Abbott, Mississauga, Canada) as described previously (10). To investigate tissue-level metabolite enrichment, brain and liver were dissected and stored at −80°C for metabolome analysis later. The whole body of zebrafish sampled at 1 h post-injection was homogenized in tris buffer, and the cortisol levels were measured as described previously (10).

For metabolomics, the tissues were homogenized in 50 mM tris buffer (pH 7.4) with a protease inhibitor cocktail (Roche Diagnostics, QC) and the metabolites were extracted using 50% ultrafiltered methanol (LC-MS grade:A454 Fisher Scientific, Canada) (1:5 dilution) as described previously (7). The extracted metabolites were analyzed using ultrahigh-performance liquid chromatography (Vanquish UHPLC, Thermo Fisher Scientific)–mass spectrometry (MS) (LC-MS; Q-Exactive HF hybrid Quadruple-orbitrap, Thermo Fisher Scientific) at the University of Calgary’s Metabolomic Research Facility (CMRF). Reversed-phase ion-pair (RPIP) liquid chromatography was utilized to screen central carbon metabolites. Retention time and mass-to-charge ratio (*m*/*z*)-based peak grouping and alignment were performed using El-Maven freeware (20). Cured peak intensities were corrected for natural isotope abundance using AccuCor (21). The identified metabolites were selected using the 80% rule (22), and the missing value imputation (MIV) due to possible limit of quantification (LOQ) was performed using missForest as described previously (23). Both AccuCor and missForest were performed using R studio. The rest of the data analysis was performed using Metaboanalyst 6.0.

### 2.4 Statistics and data presentation

Metabolites labeled with ^13^C were identified with a prefix M+(*n*), where (*n*) denotes the number of carbons in the metabolites labeled with ^13^C and expressed in percentage as mass isotopomere distribution (MID) by normalizing to the sum of all the isotopologues of a particular metabolite (24, 25). The percent labeling of different metabolites was plotted as stacked bar graphs. Effect of genotypes on each isotopologue of individual metabolite was statistically analyzed between the genotypes by using two-way analysis of variance (ANOVA) (Fisher LSD *post hoc*) utilizing the GraphPad Prism software. Since glutamine and glutamate are indistinguishable in the LC-MS due to their overlapping *m*/*z* (147.1), the data are presented as glutamine/glutamate (Gln/Glu) across the investigated tissues (26, 27). The endogenous metabolites were subjected to data processing approaches to minimize systemic bias and noise as described previously (28). Statistical univariate and multivariate analyses using Kyoto Encyclopedia of Genes and Genomes (KEGG) pathway library were performed using the Metaboanalyst 6.0 software (29). Partial least square discrimination analysis (PLS-DA) was performed for dimension reduction and clustering of metabolites between the genotypes (30). The confidentiality of the scores plots was validated using the Q2 parameter (31). If the scores plot did not pass the Q2 confidentiality, all the parent metabolites were subjected to zebrafish-specific KEGG pathway analysis. The significantly affected pathways with more than one hits were further subjected to enrichment and pathway directionality analysis. The peak intensities of the metabolites (normalized to biomass) identified within the pathways differentially affected by the cortisol treatment among the WT and knockout zebrafish were plotted separately as whisker box plots using the GraphPad prism software. These metabolites were subjected to one-way ANOVA (Tukey’s *post hoc*) when the assumptions of equal standard deviation (equal SD) were passed. When the equal SD assumptions were not met after log transformation, the metabolites were analyzed using nonparametric one-way ANOVA (Brown-Forsythe and Welch tests).

## 3 Results

### 3.1 Cortisol and glucose

**Figure 1:**
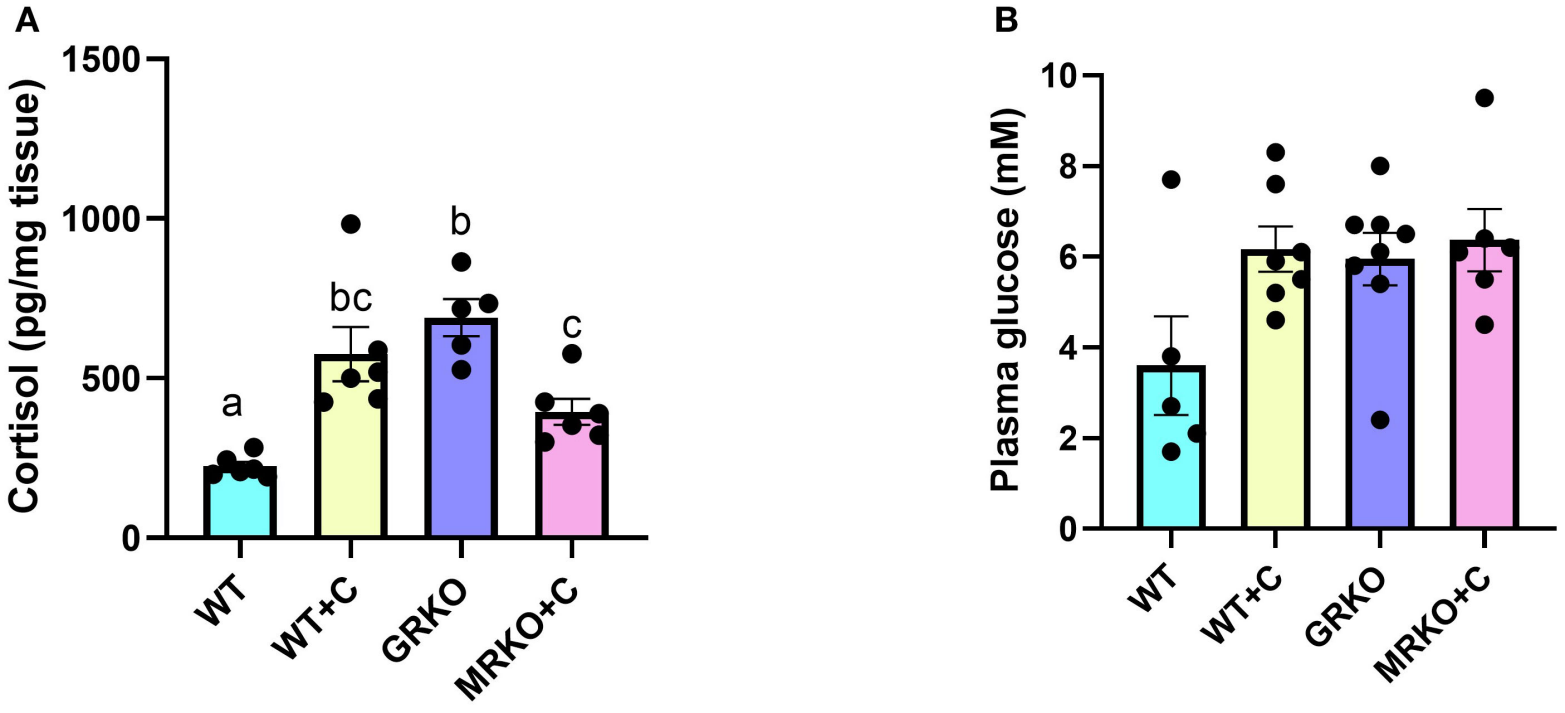
Whole-body cortisol and blood glucose levels. **(A)** Cortisol levels in whole-body (pg/mg tissue) were measured in the control (blue), cortisol-treated wild type (yellow), GRKO (purple), and MRKO (pink) zebrafish after 1 h post-injection of ^13^U-glucose following a 17-h post-waterborne cortisol exposure. **(B)** Blood glucose levels measured at 1 h post-injection of ^13^U-glucose from the treatment groups. Bars represent mean ± SEM; bars with a different letter are significantly different (one-way ANOVA, *p* < 0.05; *N* = 6). WT, wild-type control; WT+C, cortisol-treated wild type; GRKO, glucocorticoid receptor knockout (*nr3c1^−/−^*); MRKO+C, cortisol-treated mineralocorticoid receptor knockout (*nr3c2^−/−^*).

Waterborne cortisol exposure led to a 1.7- and 2.5-fold increase in the whole-body cortisol levels in the MRKO and WT zebrafish, respectively (*p* = 0.033 and 0.027, respectively, **Figure 1A**). GRKO zebrafish larvae are inherently hypercortisolemic (7), and this was also evident (threefold higher) in the present study compared to the WT zebrafish (*p* = 0.003; **Figure 1A**). The cortisol-treated MRKO zebrafish had significantly lower cortisol levels compared to the GRKO zebrafish (*p* = 0.015; **Figure 1A**). The blood glucose measured after 1 h post-U-^13^C-glucose injection was not significantly different between the treatment groups (main effect; *p* = 0.056; **Figure 1B**).

### 3.2 Labeled intermediates from glucose in the serum

**Figure 2:**
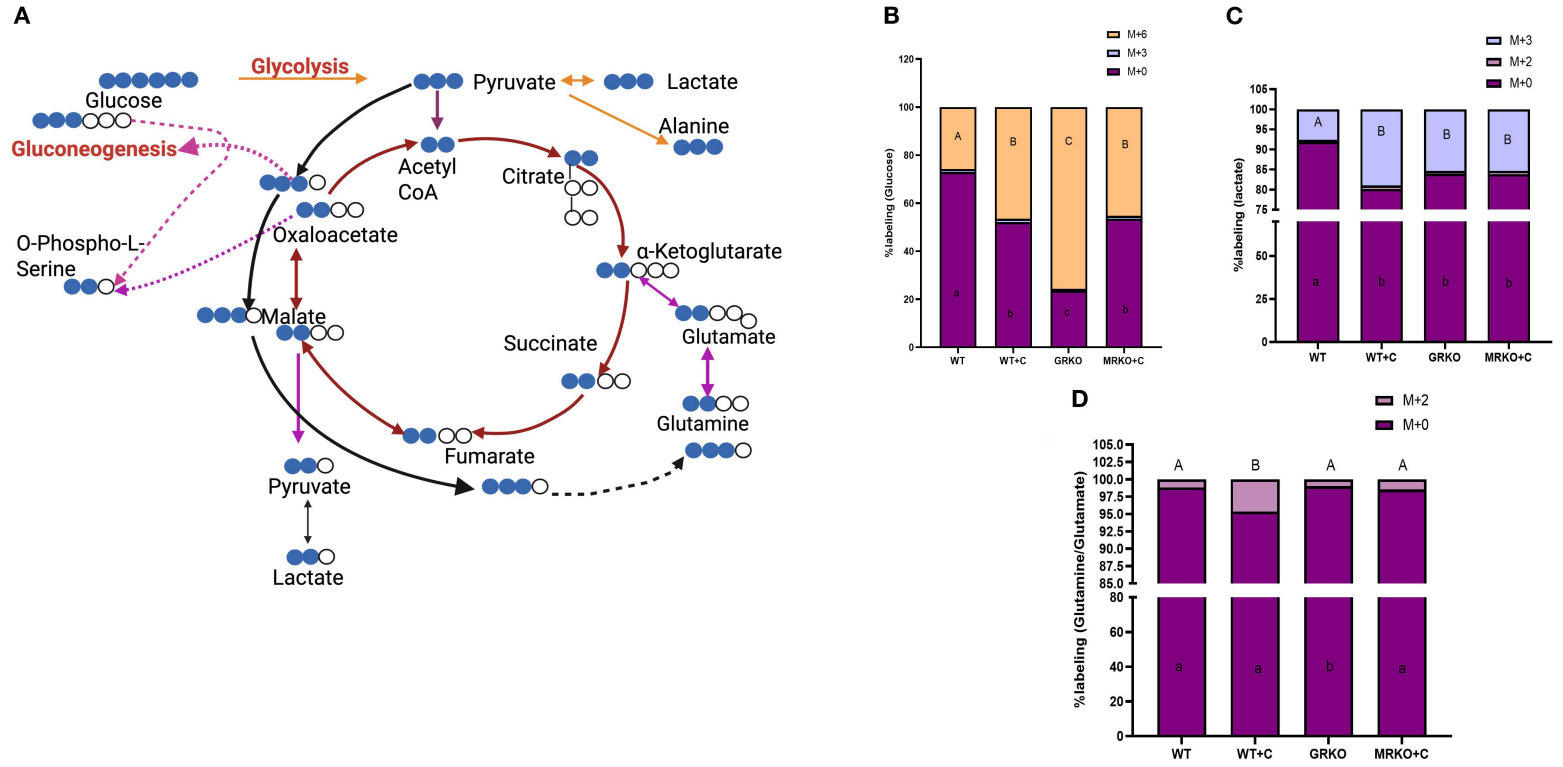
Fate of ^13^C-glucose in the serum. **(A)** Schematic showing that the transfer of ^13^C originated from the ^13^U-glucose within the glycolytic and TCA pathways. Blue circles represent ^13^C, while the white circle represents endogenous ^12^C. The red arrow indicates the carbon transfer when the pyruvate enters the TCA via acetyl CoA. The black arrow indicates the carbon transfer when the pyruvate enters the TCA via oxaloacetate. The pink arrow shows cataplerosis and anaplerosis. Dotted arrows between two metabolites indicate the presence of several intermediates in between, which were excluded for brevity. Image created in BioRender. Vijayan, M. (2025) https://BioRender.com/os6bqg0. Stacked bar graph showing the mass isotopologue distribution (MID) of glucose **(B)**, lactate **(C)**, and glutamine **(D)** measured at 1 h post-injection. Different color represents different isotopologue, expressed as percentage in respect to the total pool size (labeled and unlabeled) of the respective metabolite. The legend for each color corresponding to each isotopologue in the stacked bar is shown on the top right corner of the graph and identified as M+(*n*), where (*n*) indicates the number of ^13^C incorporated into each metabolite isotopologue. Bars with different uppercase letters (M + 6) and lowercase letters (M + 0) indicate significant difference for the respective isotopologue (two-way ANOVA, *p* < 0.05; *N* = 5). WT, wild-type control; WT+C, cortisol-treated wild type; GRKO, glucocorticoid receptor knockout (*nr3c1^−/−^*); MRKO+C, cortisol-treated mineralocorticoid receptor knockout (*nr3c2^−/−^*).

The ^13^C-labeled intermediates were identified based on the incorporation of the carbon from the U-^13^C-glucose into the central carbon metabolism as illustrated in **Figure 2A**, and described previously (24). The TCA cycle with a red arrow shows the ^13^C labeling pattern when pyruvate enters via acetyl CoA, while the black arrow shows ^13^C labeling when the pyruvate enters via oxaloacetate (32). The pink arrows show cataplerotic exit from or anaplerotic entry of TCA intermediates (24, 33). The MID of each identified labeled metabolite was calculated as described previously (25).

When looking at the serum glucose MID, ~25% of all the serum glucose isotopologues were M + 6 labeled in the WT control group at 1 h post-injection, and this was significantly different from the rest of the groups (**Figure 2B**). This M + 6 labeling was at the highest (~75%) in the GRKO group (**Figure 2B**; *p ≤* 0.0001) compared to the WT and MRKO groups. In the cortisol-treated WT zebrafish, ~45% of the serum glucose were M + 6 labeled, and this was significantly different (*p* = 0.005) compared to the WT controls but not from the MRKO group (**Figure 2B**; *p* = 0.86). This differential M + 6 labeling was also corroborated by the parent (M + 0) glucose in the serum, which was at the highest in the WT controls (~73%) and lowest in the GRKO group (~23%), while the levels in the cortisol-treated WT and the MRKO groups were similar (~50%) (**Figure 2B**). The M + 3 glucose labeling in the serum, possibly generated from gluconeogenesis (19) (**Figure 2A**), was negligible (~1%) and was consistent among all groups (**Figure 2B**).

In the cortisol-treated WT, ~20% of serum lactate was M + 3 labeled, while both GRKO and MRKO had ~15% labeling (**Figure 2C**). Higher glucose-generated lactate (M + 3) was observed irrespective of the receptor activation and was significantly higher in all the groups compared to the WT control, which only showed ~8% labeling (*p ≤* 0.01; **Figure 2C**). The M+2-labeled lactate, possibly generated from pyruvate cycling via malic enzyme (33), remained low (~1%) in all groups (**Figure 2C**). In accordance with the M + 3 lactate differential labeling, the parent lactate distribution was at the highest in the WT control (91%), while the rest of the groups showed an average of 82% (*p ≤* 0.001; **Figure 2C**). Labeled Gln/Glu, generated from glucose (the M + 2 isotopologue), was another substrate that was identified in the serum, but the majority (95% to 98%) was contributed by the parent (M + 0) Gln/Glu (**Figure 2D**). Interestingly, only the cortisol-treated WT had a ~5% distribution of M + 2 labeling among the Gln/Glu isotopologues and was significantly greater than the other groups (*p* < 0.0001; **Figure 2D**).

### 3.3 Serum endogenous metabolites

**Figure 3:**
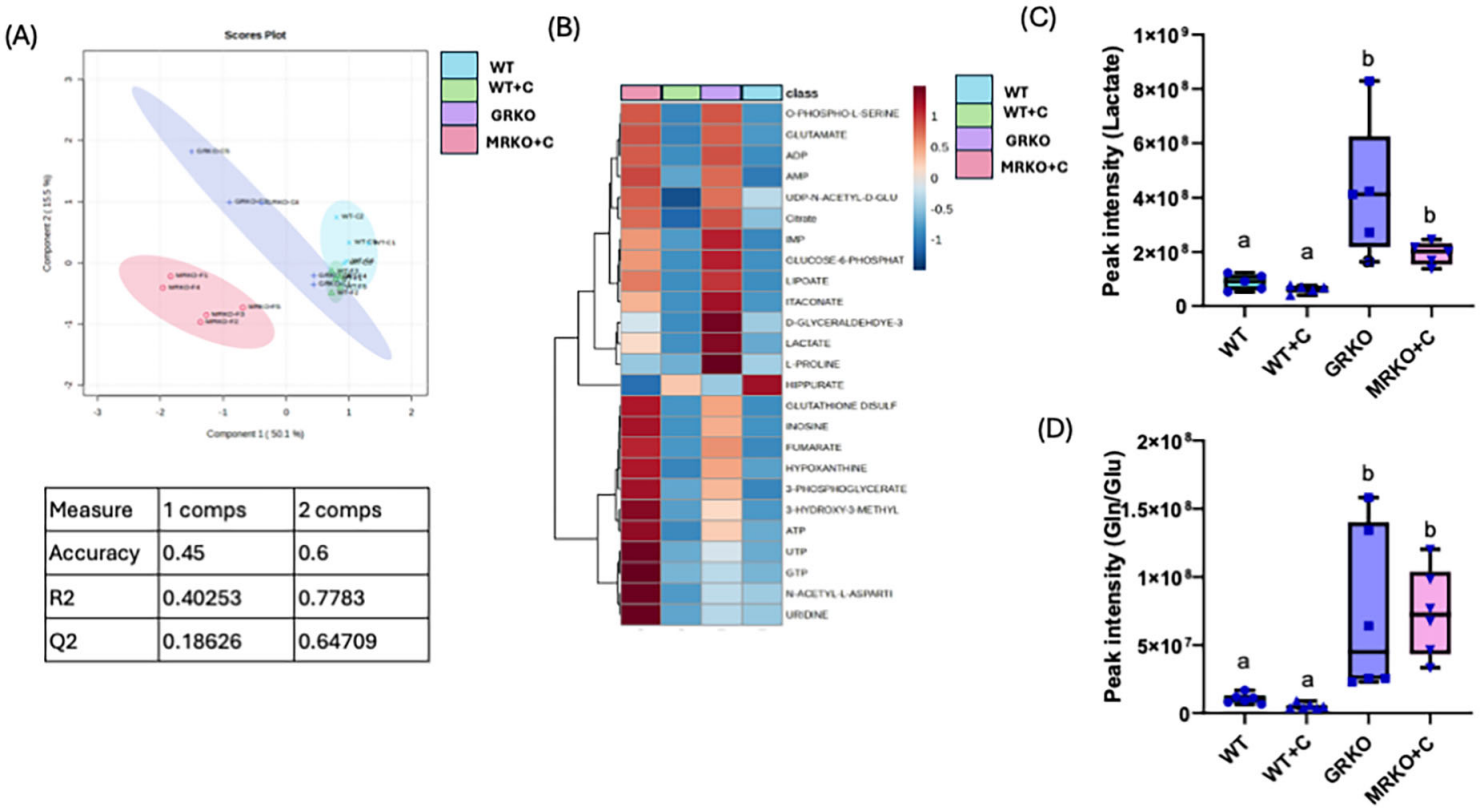
Endogenous serum metabolites. **(A)** Partial-least square discrimination analysis (PLS-DA)-generated scores scatter plot showing the metabolite profile distinction between the genotypes demonstrated by components 1 and 2. The table below contains quality check parameters including R2 (goodness of fitness scores), Q2 (goodness of prediction scores), and accuracy for components 1 and 2 from the scores plot. **(B)** Heatmap showing the relative abundances of significantly affected metabolites by cortisol and/or genotype in the serum generated by Ward’s algorithm (Metaboanalyst 6.0). Whisker box plots showing the relative abundances of endogenous lactate **(C)** and glutamine/glutamate **(D)** abundance measured as peak intensity from the liquid chromatography–mass spectrometry (LC/MS) spectra of the control WT (blue), cortisol-treated WT (yellow), GRKO (purple), and MRKO (pink) serum. Bars with a different letter indicate significant difference (one-way ANOVA, *p* < 0.05; *N* = 6). WT, wild-type control; WT+C, cortisol-treated wild type; GRKO, glucocorticoid receptor knockout (*nr3c1^−/−^*); MRKO+C, cortisol-treated mineralocorticoid receptor knockout (*nr3c2^−/−^*); Gln, glutamine; Glu, glutamate.

Serum metabolites were subjected to PLS-DA dimension reduction analysis and the scores plot showed a clear distinction between the GRKO and MKRO group with some overlapping between the control and cortisol-treated WT groups (**Figure 3A**). Although component 1 demonstrated 50% variability between the treatment groups, the Q2 confidentiality parameter was only 0.19 (**Figure 3A**) (Q2 > 0.5 shows high confidentiality; 34). However, component 2 explained 15% of the variability with high confidentiality (Q2 = 0.65). Moreover, when the global parent metabolites were subjected to one-way ANOVA (with Tukey’s HSD correction for multiple comparison), 23 metabolites were significantly affected between the investigated group. The heatmap shows the general contrast among the significantly affected metabolites between the groups, and the color range reflects the relative abundance (**Figure 3B**). The differentially regulated metabolite abundance can be distinctly observed between the GRKO and MRKO groups compared to the WT in the heatmap (**Figure 3B**). The endogenous lactate content in the serum was not different between the WT and cortisol-treated WT fish, but they were significantly higher in the GRKO and MRKO groups compared to the other groups (*p* = 0.004; **Figure 3C**). The relative abundance of endogenous Gln/Glu in the serum mimicked that of lactate with higher abundance in the GRKO and MRKO groups compared to the WT control (*p* = 0.0003 and *p* < 0.0001, respectively) and WT cortisol-treated groups (*p* < 0.0001; **Figure 3D**). The serum Gln/Glu abundance was not significantly different between the control and cortisol-treated WT (*p* = 0.13; **Figure 3D**).

### 3.4 Labeled intermediates from glucose in the liver

**Figure 4:**
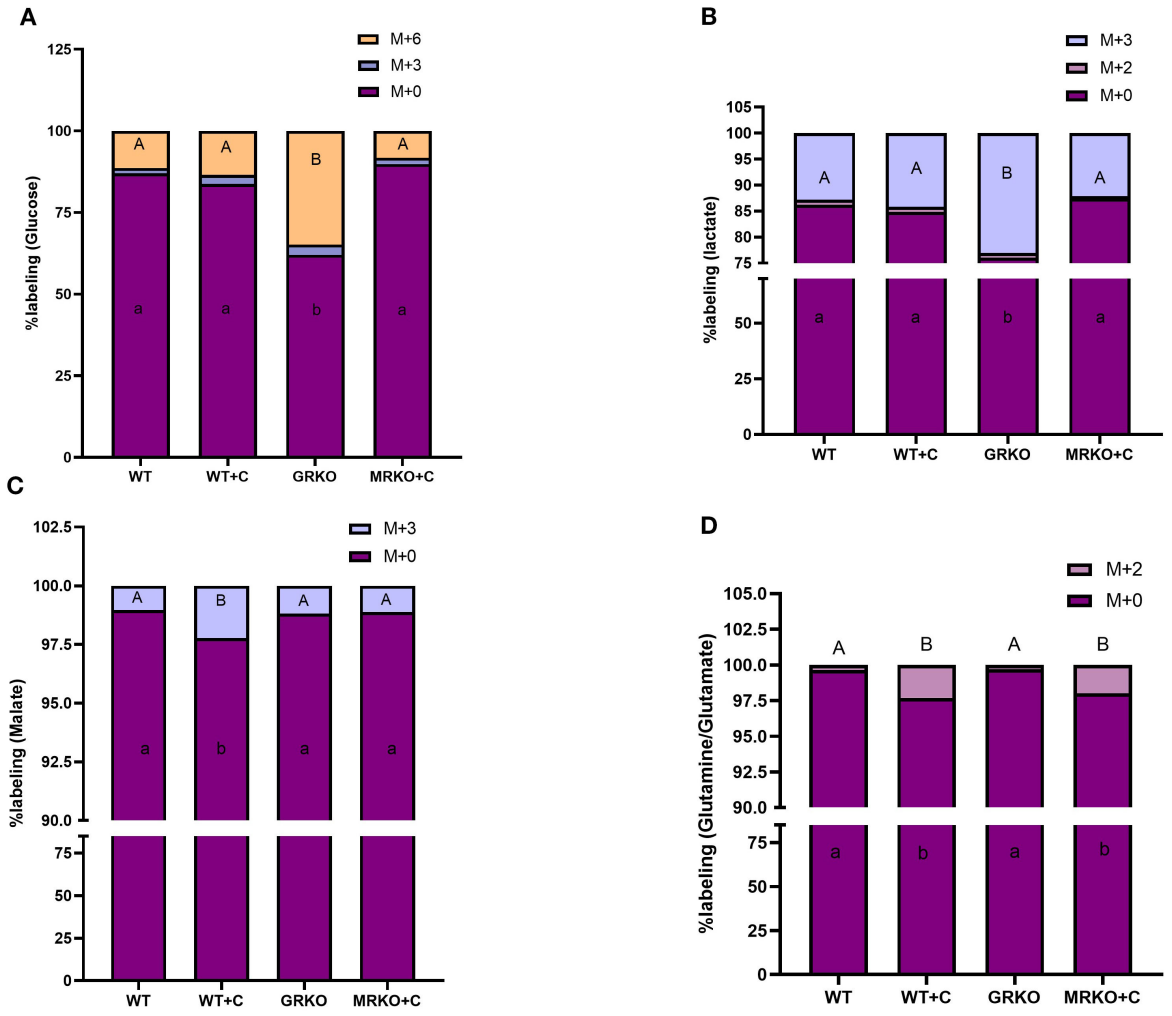
Fate of ^13^C-glucose in the liver. Stacked bar graph showing the mass isotopologue distribution (MID) of glucose **(A)**, lactate **(B)**, malate **(C)**, and glutamine/glutamate **(D)** measured at 1 h post-injection. Different color represents a different isotopologue, expressed as percentage in respect to the total pool size (labeled and unlabeled) of the respective metabolite. The legend for each color corresponding to each isotopologue in the stacked bar is shown on the top right corner of the graph and identified as M+(*n*), where (*n*) indicates the number of ^13^C incorporated into each metabolite isotopologue. Bars with different uppercase letters (M + 6 for glucose; M + 3 for malate and lactate; M + 2 for glutamine/glutamate) and lowercase letters (M + 0) indicate significant difference for the respective isotopologue (two-way ANOVA, *p* < 0.05; *N* = 5). WT, wild-type control; WT+C, cortisol-treated wild type; GRKO, glucocorticoid receptor knockout (*nr3c1^−/−^*); MRKO+C, cortisol-treated mineralocorticoid receptor knockout (*nr3c2^−/−^*).

In the liver, glucose MID representation showed that the GRKO had 25% higher distribution of M+6-labeled glucose compared to the rest of the groups (*p* < 0.0001; **Figure 4A**). The M + 3 glucose, possibly generated from gluconeogenesis (19; **Figure 2A**), showed an average distribution of 2.5% and was not significantly different between the groups (*p* = 0.92; **Figure 4A**). The parent glucose distribution was at the lowest in the GRKO group (62%; **Figure 4A**), while the rest of the group showed an average distribution of ~86% and were significantly higher than the GRKO group (*p* < 0.0001; **Figure 4A**). Lactate (M + 3), the glycolytic product of M + 6 glucose distribution, was almost doubled in the GRKO group compared to the rest of the groups (~13%, *p* < 0.0001; **Figure 4B**). The parent M + 0 lactate distribution was significantly lower in the GRKO group compared to all other groups and corresponded with the elevated M + 3 labeling seen in that group (*p* < 0.0001; **Figure 4B**). The M + 2 lactate distribution was <1% labeling in all groups (**Figure 4B**). The liver MID showed that the TCA intermediate M + 3 malate, derived possibly from pyruvate anaplerosis (24) was twofold higher in the cortisol-treated WT compared to the rest of the treatment groups (*p* = 0.01; **Figure 4C**). The parent M + 0 malate distribution was significantly lower in the cortisol-treated WT liver compared to the rest of the groups (**Figure 4C**; *p* = 0.016). Both the cortisol-treated WT and MRKO liver exhibited ~2% M + 2 Gln/Glu distribution, a cataplerotic product of TCA intermediate α-ketoglutarate (α-KG; **Figure 2A**), compared to the ~0.2% distribution in the control and GRKO liver (*p* = 0.006 and *p* = 0.02, respectively; **Figure 4D**). The M + 0 Gln/Glu showed a lower distribution in the cortisol-treated WT and MRKO groups, compared to the control and GRKO groups (**Figure 4D**). The liver M + 3 alanine distribution, derived from labeled glucose, remained approximately 7% across all groups (**Supplementary Figure S1**). Since the labeled metabolite distribution was not affected across the genotypes, the parent M + 0 alanine distribution showed no significant change between the groups (**Supplementary Figure S1**).

### 3.5 Liver endogenous metabolites

**Figure 5:**
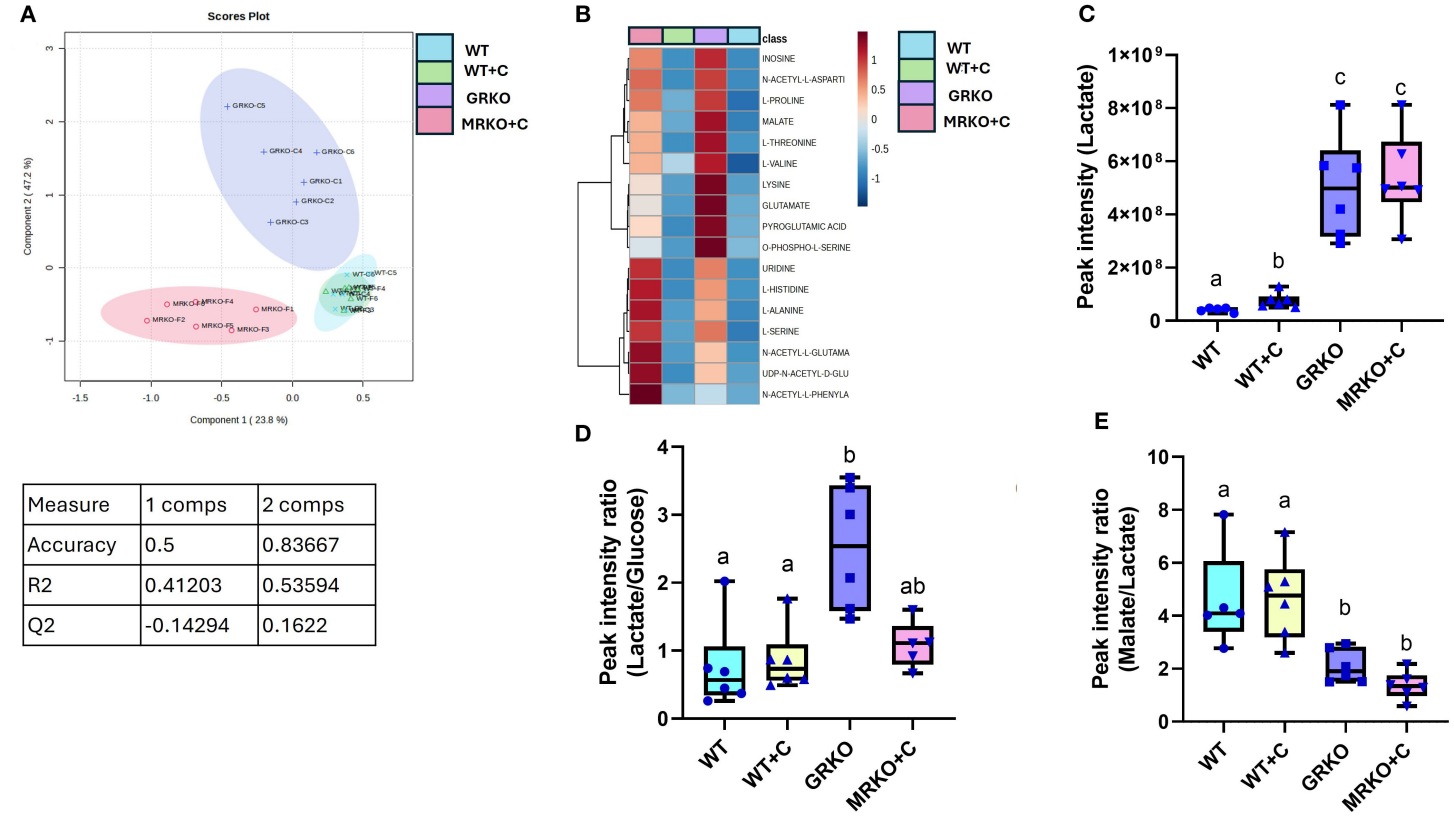
Endogenous liver metabolites **(A)** Partial-least square discrimination analysis (PLS-DA)-generated scores scatter plot showing the metabolite profile distinction between the genotypes demonstrated by components 1 and 2. The table below contains quality check parameters including R2 (goodness of fitness scores), Q2 (goodness of prediction scores), and accuracy for components 1 and 2 from the scores plot. **(B)** Heatmap showing the relative abundances of significantly affected metabolites by cortisol and/or genotype in the liver generated by Ward’s algorithm (Metaboanalyst 6.0). **(C)** Whisker box plots showing the endogenous lactate relative abundance measured from the liquid chromatography–mass spectrometry (LC/MS) spectral peak intensity normalized to the liver biomass measured from the WT control (blue), WT cortisol (yellow), GRKO (purple) and MRKO (pink) liver at 1 h post-injection. **(D)** Whisker box plot showing the relative rate of lactate production per unit of endogenous glucose within the liver. Expressed as lactate-to-glucose ratio calculated by dividing the normalized relative abundances of lactate by glucose. **(E)** Whisker box plot showing the relative rate of malate generated per unit of endogenous lactate. Expressed as malate-to-lactate ratio calculated by dividing the normalized relative abundances of malate by lactate. Bars with a different letter indicate significant difference (one-way ANOVA, *p* < 0.05; *N* = 6). WT, wild-type control; WT+C, cortisol-treated wild type; GRKO, glucocorticoid receptor knockout (*nr3c1^−/−^*); MRKO+C, cortisol-treated mineralocorticoid receptor knockout (*nr3c2^−/−^*).

When all the identified parent metabolites in the liver were subjected to dimension reduction PLS-DA, the GRKO and MRKO groups showed a clear separation with components 1 and 2 depicting 23.8% and 47.2% variability, respectively (**Figure 5A**). Since the confidentiality Q2 scores did not meet the threshold level (**Figure 5A**), we subjected all the 32 identified metabolites to one-way ANOVA (Tukey HSD *post hoc*) analysis and KEGG pathway analysis specific to zebrafish using Metaboanalyst 6.0 as described previously (30). There were 17 metabolites, which showed a significant enrichment pattern across the WT, GRKO, and MRKO liver and are displayed as a heatmap (**Figure 5B**). Pathway enrichment analysis was first performed in the WT to establish the effect of cortisol, and then both GRKO and MRKO were compared separately with the cortisol-treated WT. The 10 most affected pathways in the liver are listed in **Table 1**. A cutoff value of 0.1 impact score was considered a significant metabolite for any designated pathway (35). Furthermore, GRKO and MRKO showed a significant difference from the cortisol-treated WT for all the 10 pathways (**Table 1**). The glycolysis pathway, with 3 enriched metabolites, was significantly different in the GRKO and MRKO groups compared to the cortisol-treated WT separately. Since labeled glycolytic intermediates were also differentially regulated in the knockouts, we looked at the endogenous metabolite pool size and the production/consumption pathways.

**Table 1:** List of metabolic pathways in the liver significantly different between the treatments/genotypes when separately analyzed against cortisol-treated wild type.

Pathway	Match	Impact	WT vs. WT+C	WT+C vs. GRKO	WT+C vs. MRKO+C
			FDR	FDR	FDR
Pyruvate metabolism	4/23	0.0283	0.013057	7.3236E-4	0.0063868
Valine, leucine, and isoleucine biosynthesis	2/40	0	8.4875E-5	0.028316	0.0075825
Histidine metabolism	2/27	0.22449	0.023355	6.1139E-4	2.234E-4
Glycolysis	3/26	0.16435	0.065089	0.0034033	0.0026807
Glycine, serine, and threonine metabolism	3/33	0.25974	0.06595	0.009486	4.4521E-4
Alanine, aspartate, and glutamate metabolism	6/27	0.33989	0.16641	0.009486	2.234E-4
Arginine and proline metabolism	6/27	0.33989	0.16641	0.009486	2.234E-4
Citrate cycle (TCA cycle)	4/35	0.28554	0.14515	0.030204	0.0072971
Purine metabolism	2/71	0.05593	0.18257	0.009486	0.0052707
Pyrimidine metabolism	1/41	0.04565	0.10061	0.0087307	0.0091192

The “Match” column shows the number of metabolites identified in respect to the total number of metabolites involved in each pathway. The “Impact” column shows the numerical representation of matched metabolites’ importance and centrality of a specific metabolic pathway, normalized to the sum of all the metabolites within the pathway. FDR, false discovery rate; WT, wild-type control; WT+C, cortisol-treated wild type; GRKO, glucocorticoid receptor knockout (*nr3c1^−/−^*); MRKO+C, cortisol-treated mineralocorticoid receptor knockout (*nr3c2^−/−^*).

In comparison to the MID (**Figure 4**), the liver endogenous lactate pool size was significantly higher in both the GRKO and MRKO groups compared to the WT groups (*p* < 0.0001; **Figure 5C**). Cortisol treatment to the WT increased the lactate pool from the control (*p* = 0.038; **Figure 5C**) but lower than the GRKO and MRKO groups. An increase in relative abundance of this metabolite could be from either enhanced production or a decreased consumption within the metabolic pathway (25). Therefore, to get some idea of potential liver lactate turnover, we calculated the ratio of lactate to glucose (suggests production) and malate (the subsequent intermediate) to lactate (suggests consumption), as shown previously (25). The lactate-to-D-glucose ratio was elevated only in the GRKO liver compared to the control and cortisol-treated WT (*p* = 0.0007 and *p* = 0.006; **Figure 5D**), but not the MRKO group (*p* = 0.06; **Figure 5D**). In both the GRKO and MRKO groups, the malate-to-lactate ratio was significantly lower compared to the control (*p* = 0.01 and 0.0001, respectively) and cortisol-treated WT groups (*p* = 0.008 and <0.0001, respectively; **Figure 5E**). There were no significant differences between the malate-to-lactate ratios between the control and cortisol-treated WT or between the MRKO and GRKO groups (**Figure 5E**).

**Figure 6:**
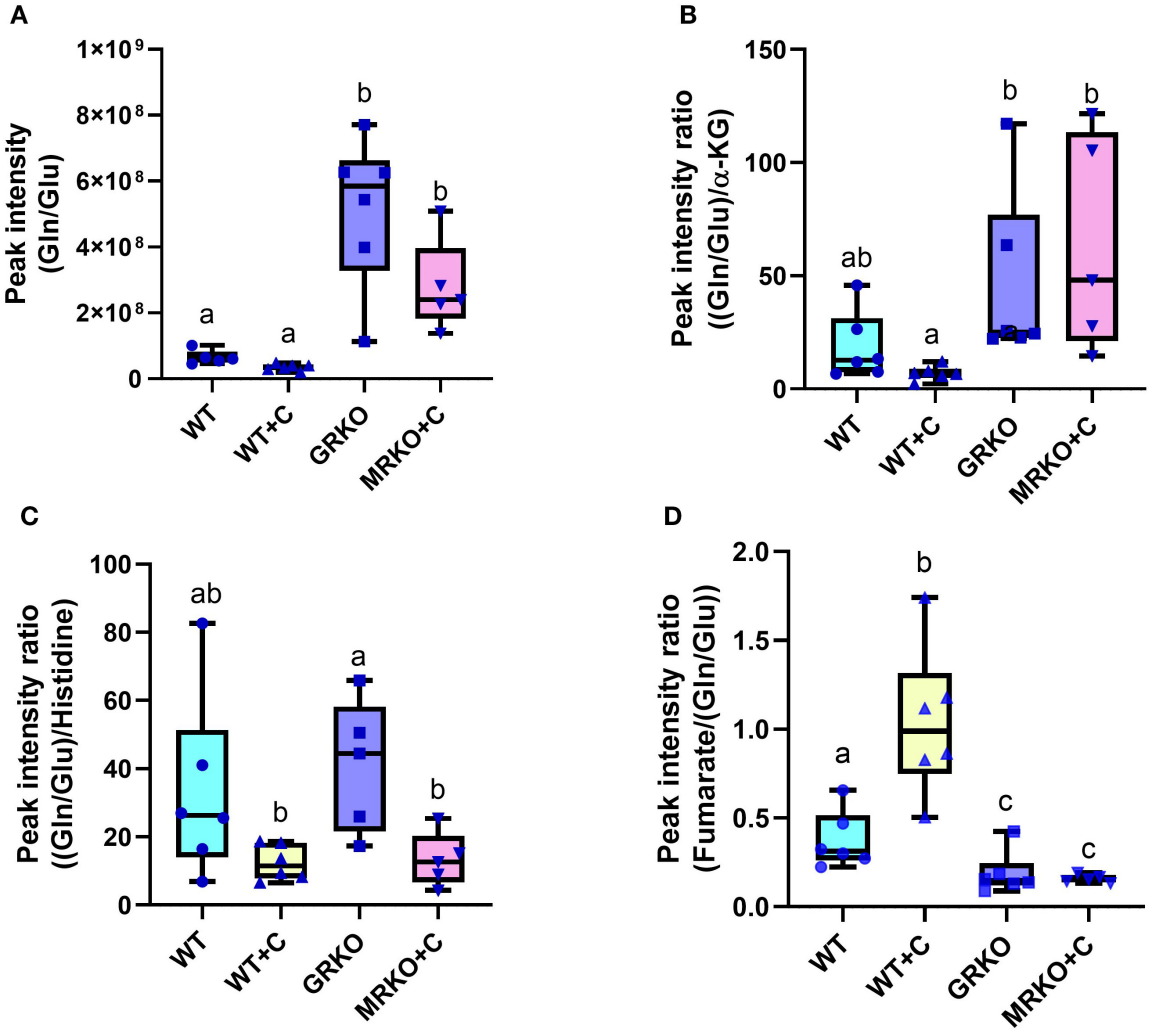
Liver endogenous glutamine/glutamate metabolism. **(A)** Whisker box plots showing the endogenous glutamine/glutamate relative abundance measured from the liquid chromatography–mass spectrometry (LC/MS) spectral peak intensity normalized to the liver biomass measured from the WT control (blue), WT cortisol (yellow), GRKO (purple), and MRKO (pink) liver at 1 h post-injection. **(B)** Whisker box plot showing the relative rate of glutamine/glutamate production from α-ketoglutarate (α-KG) within the liver. Expressed as Gln/Glu-to-α-KG ratio calculated by dividing the normalized relative abundances of glutamine/glutamate by α-ketoglutarate. **(C)** Whisker box plot showing the relative rate of glutamine/glutamate production from histidine metabolism within the liver. Expressed as Gln/Glu-to-histidine ratio calculated by dividing the normalized relative abundances of glutamine/glutamate by histidine. **(D)** Whisker box plot showing the relative rate of glutamine/glutamate consumption into the TCA cycle in the liver. Expressed as fumarate-to Gln/Glu ratio calculated by dividing the normalized relative abundances of fumarate by glutamine/glutamate. Different lowercase letters indicate significant difference. Bars with a different letter indicate significant difference (one-way ANOVA, *p* < 0.05; *N* = 6). WT, wild-type control; WT+C, cortisol-treated wild type; GRKO, glucocorticoid receptor knockout (*nr3c1^−/−^*); MRKO+C, cortisol-treated mineralocorticoid receptor knockout (*nr3c2^−/−^*); Gln, glutamine; Glu, glutamate; α-KG, α-ketoglutarate.

Another pathway that was significantly affected across all comparisons was histidine metabolism (**Table 1**). Histidine metabolism with two matched intermediates (histidine and Gln/Glu) showed a cumulative impact score of 0.224. Since the labeled Gln/Glu distribution showed a distinct pattern, we further investigated the endogenous Gln/Glu status. An increased Gln/Glu pool size was observed in both the GRKO and MRKO liver compared to the other two groups (*p* = 0.01; **Figure 6A**). To investigate, if this was due to an increased production, we calculated the Gln/Glu-to-α-ketoglutarate ratio and Gln/Glu-to-histidine ratio (36), while for the consumption pathway, we calculated the fumarate-to-Gln/Glu ratio (24, 37, 38). Both the GRKO and MRKO liver showed a significantly higher Gln/Glu-to-α-ketoglutarate ratio compared to the cortisol-treated WT (*p* = 0.001; **Figure 6B**). The Gln/Glu-to-histidine ratio was significantly elevated in the GRKO liver compared to the MRKO and cortisol-treated WT (*p* = 0.04; **Figure 6C**), but not in the WT controls. On the consumption side, both the GRKO and MRKO liver had a reduced fumarate-to-Gln/Glu ratio compared to the control and cortisol-treated WT groups (*p* = 0.02 and *p* = 0.01, respectively; **Figure 6D**). Moreover, cortisol-treated WT showed a significantly higher fumarate-to-Gln/Glu ratio compared to the rest of the groups (*p* = 0.001 and *p* < 0.0001; **Figure 6D**).

### 3.6 Labeled glycolytic intermediates from glucose in the brain

**Figure 7:**
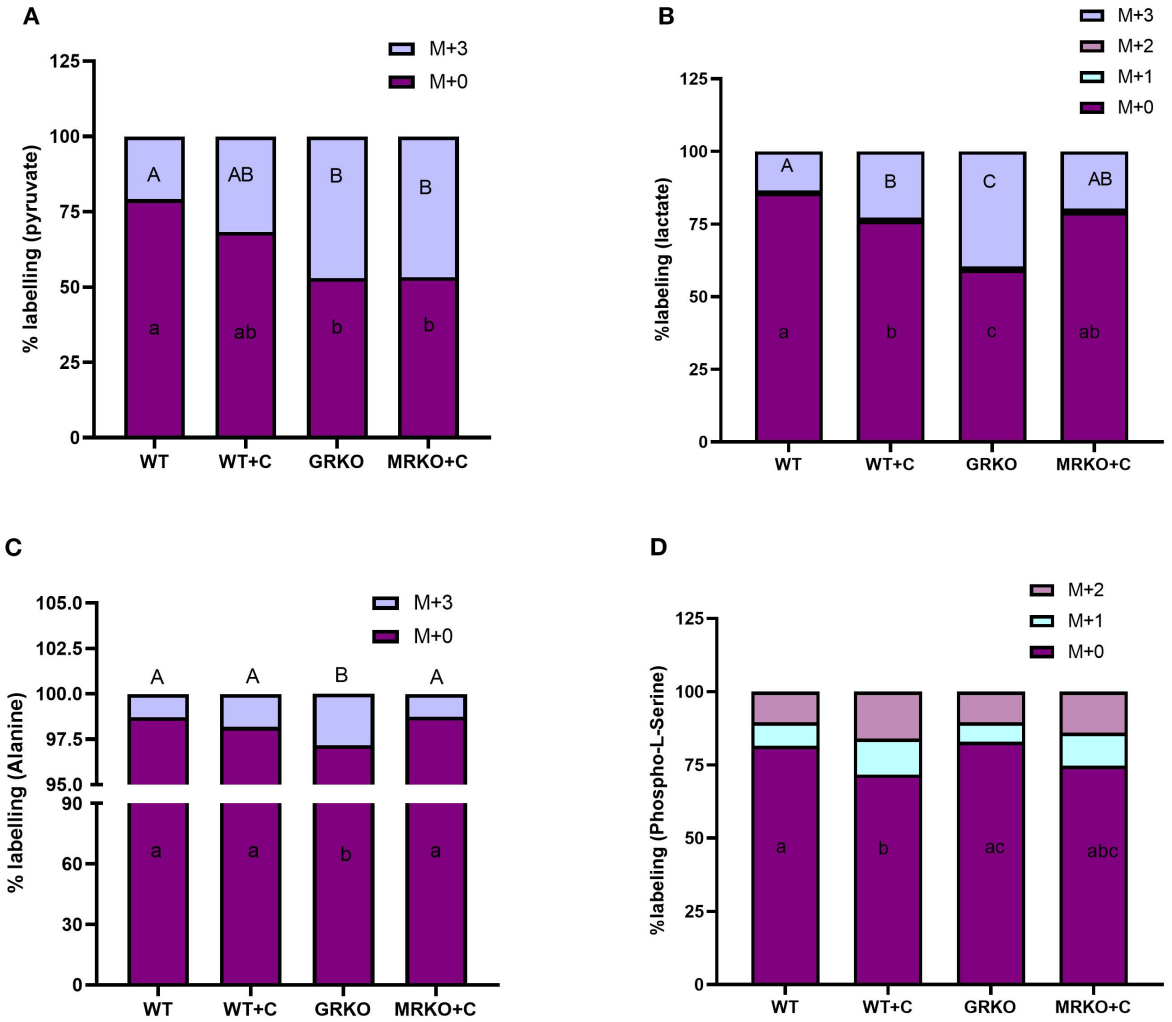
Fate of ^13^C glucose within the glycolytic pathway in the brain. Stacked bar graph showing the mass isotopologue distribution (MID) of pyruvate **(A)**, lactate **(B)**, alanine **(C)**, and phosphoserine **(D)** measured at 1 h post-injection. Different color represents a different isotopologue, expressed as percentage in respect to the total pool size (labeled and unlabeled) of the respective metabolite. The legend for each color corresponding to each isotopologue in the stacked bar is shown on the top right corner of the graph and identified as M+(*n*), where (*n*) indicates the number of ^13^C incorporated into each metabolite isotopologue. Bars with different uppercase letters (M + 3) and lowercase (M + 0) letters indicate significant difference for the respective isotopologue (two-way ANOVA, *p* < 0.05; *N* = 5). WT, wild-type control; WT+C, cortisol-treated wild type; GRKO, glucocorticoid receptor knockout (*nr3c1^−/−^*); MRKO+C, cortisol-treated mineralocorticoid receptor knockout (*nr3c2^−/−^*).

The brain exhibits the highest metabolic demand per unit mass compared to any tissue (39). Consequently, most of the ^13^C-labeled glucose was utilized in the brain tissue within 1 h post-injection. However, M + 3 pyruvate, the glycolytic end product of labeled glucose, was elevated in both the GRKO and MRKO groups (~46%) and was significantly different from the WT control (*p* = 0.003; **Figure 7A**). The cortisol-treated WT had ~30% of M + 3 pyruvate, and it was not significantly different from the WT control (~20%) (*p* = 0.2; **Figure 7A**) and the MRKO and GRKO groups (*p* = 0.07; **Figure 7A**). The parent M + 0 pyruvate distribution in the brain was significantly lower in the GRKO and MRKO groups compared to the WT control (*p* < 0.0001 and *p* = 0.001, respectively; **Figure 7A**). M + 3 lactate generated from pyruvate was significantly higher in the GRKO brain compared to the rest of the groups (~40%, *p* < 0.0001; **Figure 7B**). The cortisol-treated WT exhibited the second highest M + 3 lactate (22%) distribution and was significantly higher than the WT control (13%, *p* = 0.01; **Figure 7B**), but lower than GRKO (*p* < 0.0001) and not different from MRKO (*p* = 0.4; **Figure 7B**). MRKO showed ~19% labeling with no difference from the WT groups but was lower than the GRKO group (*p* < 0.0001; **Figure 7B**). The distribution of M + 2 and M + 1 lactate was not significantly different between the groups with a mean labeling of 0.25% and 0.8%, respectively (**Figure 7B**). The parent M + 0 lactate distribution corresponded to M+3 labeling, with GRKO showing the lowest distribution (*p* < 0.0001; **Figure 7B**), while the cortisol-treated WT showed the second lowest distribution and was significantly different from the WT control (*p* = 0.01) and GRKO (*p* < 0.0001), but not from MRKO (*p* = 0.4; **Figure 7B**). Alanine is another glycolytic product generated from the labeled glucose in the brain. The M + 3 distribution was significantly higher in the GRKO brain (~3%), which was more than twofold higher than the average distribution of the control and MRKO groups (*p* = 0.0005; **Figure 7C**) and 1.5-fold higher than the cortisol-treated WT (*p* = 0.02; **Figure 7C**). The parent M + 0 alanine distribution was significantly lower in the GRKO group compared to the rest of the groups and corresponded with the labeled alanine distribution (**Figure 7C**). “Phosphorylated pathway” is involved in L-serine biosynthesis from glycolytic intermediate 3-phosphoglycerate (40). We did observe the M + 2- and M + 1-labeled isotopologues of phosphoserine, a final rate-limiting intermediate of L-serine biosynthesis (41). The M + 2 and M + 1 phosphoserine labeling was not significantly different among the treatment groups (**Figure 7D**). However, the parent M + 0 phosphoserine was significantly lower in the cortisol-treated WT brain with only 71% labeling, compared to ~80% labeling in the WT control and GRKO groups (*p* = 0.03 and 0.01, respectively; **Figure 7D**). The MRKO with 74% distribution was not significantly different from the rest of the groups (**Figure 7D**). The cortisol-treated WT brain showed a 12% and 15.9% of M + 1 and M + 2 labeling, respectively, compared to ~ 7% and ~10% labeling in the WT control and GRKO brains, respectively (**Figure 7D**).

### 3.7 Labeled TCA cycle intermediates from glucose in the brain

**Figure 8:**
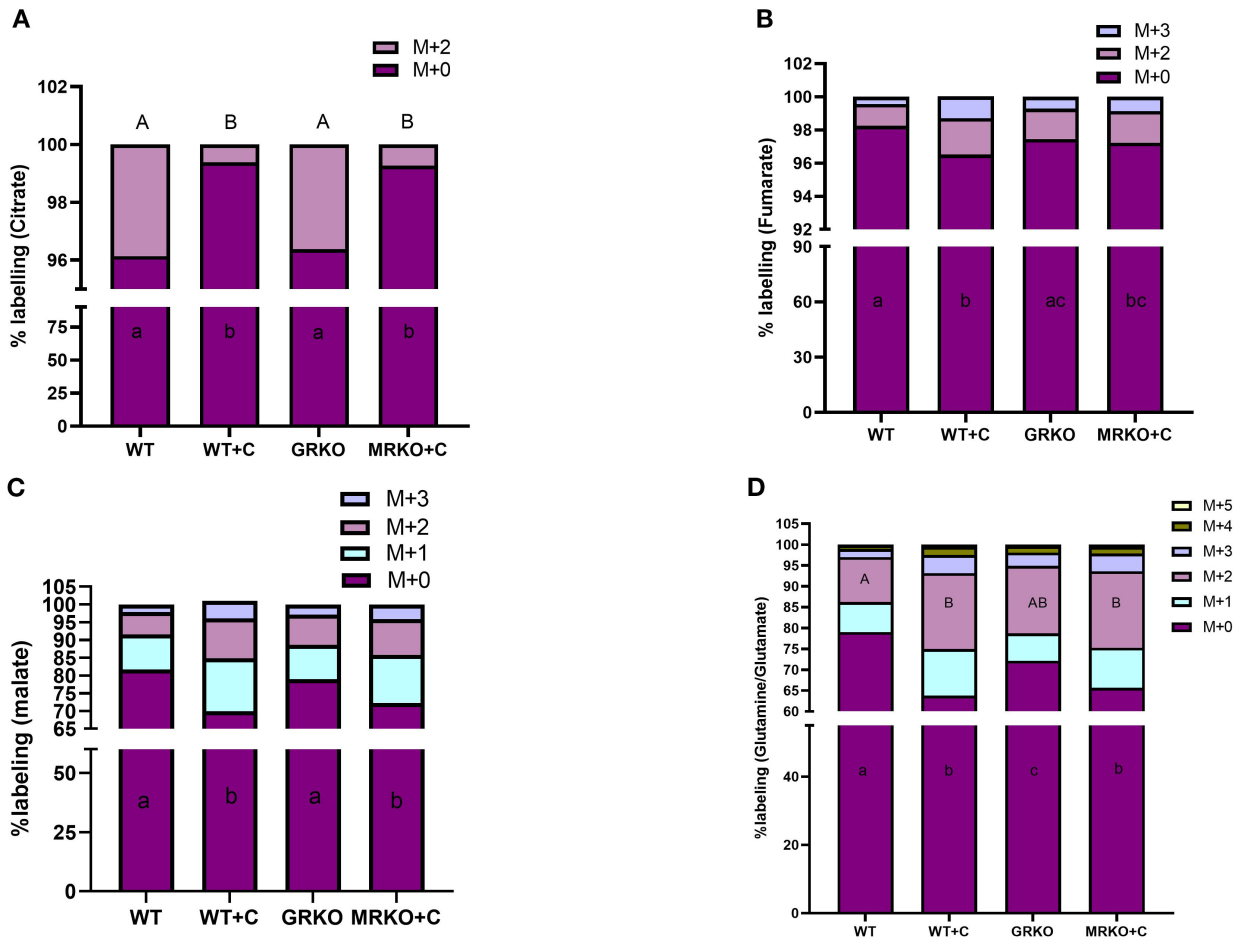
Fate of ^13^C glucose within the TCA pathway in the brain. Stacked bar graph showing the mass isotopologue distribution (MID) of different isotopologues of citrate **(A)**, fumarate **(B)**, malate **(C)**, and glutamine/glutamate **(D)** measured at 1 h post-injection. Different color represents a different isotopologue, expressed as percentage in respect to the total pool size (labeled and unlabeled) of the respective metabolite. The legend for each color corresponding to each isotopologue in the stacked bar is shown on the top right corner of the graph and identified as M+(*n*), where (*n*) indicates the number of ^13^C incorporated into each metabolite isotopologue. Bars with different uppercase letters (M + 2) and lowercase (M + 0) letters indicate significant difference for the respective isotopologue (two-way ANOVA, *p* < 0.05; *N* = 5). WT, wild-type control; WT+C, cortisol-treated wild type; GRKO, glucocorticoid receptor knockout (*nr3c1^−/−^*); MRKO+C, cortisol-treated mineralocorticoid receptor knockout (*nr3c2^−/−^*).

The GRKO and MRKO brain showed a significantly lower M + 2 citrate distribution (~0.7%) in contrast to ~4% with the WT control and cortisol-treated WT groups (*p* < 0.0001; **Figure 8A**). Hence, almost all the citrate MIDs (~99.25%) were composed of the parent citrate in both the GRKO and MRKO groups compared to the WT groups (**Figure 8A**). Despite the low M + 2 citrate labeling in the mutants, there were no significant differences in the α-KG (**Supplementary Figure S2A**) or succinate isotopologue labeling (**Supplementary Figure S2B**) among the treatment groups in the present study. The M + 2 α-KG distribution was ~17% labeling, while the distribution for M + 1 and the parent M + 0 was approximately 10% and 70%, respectively, in all groups (**Supplementary Figure S2A**). The M + 2, M + 1, and M + 0 succinate had a mean distribution of 17.5%, 16.7%, and 66%, respectively, in all groups (**Supplementary Figure S2B**). The cortisol-treated WT zebrafish brain showed an almost significant increase in the distribution of M + 3 and M + 2 fumarate labeling with 1.3% and 2.2% in comparison to the WT control, which showed only 0.44% and 1.3%, respectively (*p* = 0.052; **Figure 8B**). The GRKO and MRKO brains with 0.8% and 1.8% distribution of M + 2 and M + 1 isotopologues were not different from the rest of the groups (**Figure 8B**). The parent fumarate distribution (~96%) was significantly lower in the cortisol-treated WT compared to the control and GRKO (*p* = 0.0003 and 0.03, respectively) but not the MRKO (*p* = 0.1; **Figure 8B**) group. The next TCA intermediate malate showed M + 0-, M + 1-, M + 2-, and M+3-labeled isotopologues. The M + 2 and M + 1 malate distribution in the cortisol-treated WT showed a relatively higher but not significant labeling with 11% and 15%, in contrast to the 6.3% and 10% in the WT control, respectively (*p* = 0.08 and *p* = 0.075, respectively; **Figure 8C**). However, these relatively higher isotopologues also reflected in the parent M + 0 distribution in the cortisol-treated WT, which was 70% and significantly lower in comparison with the WT control and GRKO groups (~80%, *p* < 0.0001 and *p* = 0.002; **Figure 8C**). The MRKO group with 10% and 13.5% distribution of M + 2 and M + 1 labeling, respectively, was not significantly different across the treatments. However, these relative increases in the MRKO group affected the parent malate distribution significantly (72%) compared to the WT control and GRKO (*p* = 0.003 and *p* = 0.02, respectively), but not from the cortisol-treated WT (*p* = 0.4, **Figure 8C**). The M + 3 malate with a mean labeling of 3.5% was not significantly different among any groups (**Figure 8C**). Glutamine is the most abundant amino acid in the brain and a critical precursor of glutamate, a vital neurotransmitter (42, 43). Glutamate is generated by the cataplerotic activity of α-KG and then converted to glutamine (**Figure 2A**). In our study, the isotopomeres M + 0 through M + 5 were observed at an average distribution of ~70%, 8%, 15%, 3.5%, 1.5%, and 0.4%, respectively, in all groups (**Figure 8D**). However, not all the isotopomere distribution was consistent between the treatment groups. The cortisol-treated WT brain showed the lowest parent Gln/Glu distribution with 63% and was significantly different from the WT control (79%, *p* < 0.0001) and GRKO (72%, *p* = 0.005) but not from MRKO (65%, *p* = 0.5; **Figure 8D**). Similarly, the second most abundant isotopologue was M + 2 Gln/Glu. Both cortisol-treated WT and MRKO brain showed an ~18% labeling and was significantly higher from the WT control (10.8%, *p* = 0.01), but not from GRKO (16%, *p* = 0.5; **Figure 8D**). The M + 1, M + 3, M + 4, and M + 5 Gln/Glu distribution was not affected across the groups, showing a mean labeling of 8%, 3.5%, 1.5%, and 0.4%, respectively (**Figure 8D**).

### 3.8 Brain endogenous metabolites

**Figure 9:**
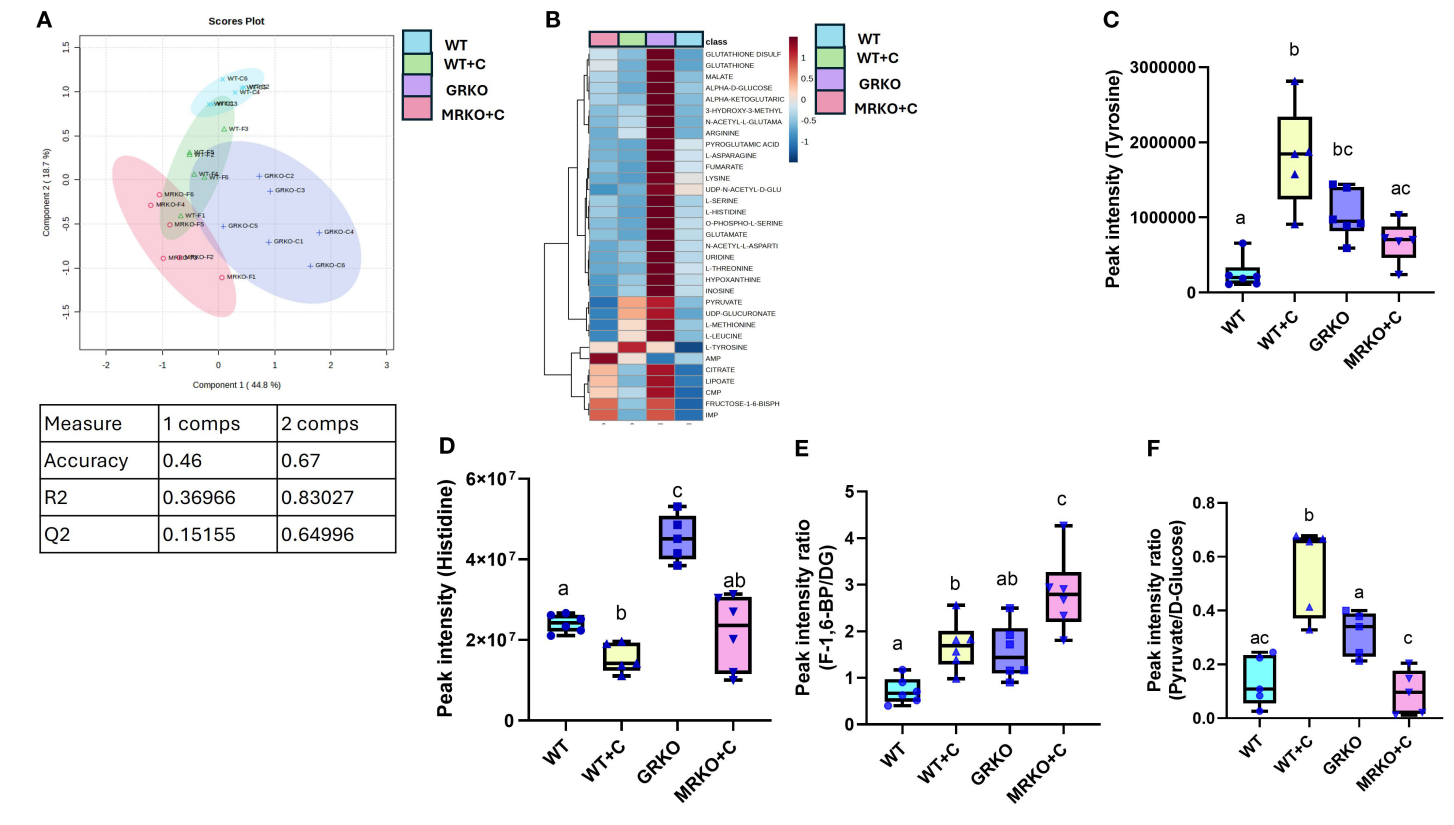
Endogenous brain metabolites. **(A)** Partial-least square discrimination analysis (PLS-DA)-generated scores scatter plot showing the metabolite profile distinction between the genotypes demonstrated by components 1 and 2. The table below contains quality check parameters including R2 (goodness of fitness scores), Q2 (goodness of prediction scores), and accuracy for components 1 and 2 from the scores plot. **(B)** Heatmap showing the relative abundances of significantly affected metabolites by cortisol and/or genotype in the brain generated by Ward’s algorithm (Metaboanalyst 6.0). Whisker box plots showing the tyrosine **(C)** and histidine **(D)** relative abundance measured from the liquid chromatography–mass spectrometry (LC/MS) spectral peak intensity normalized to the brain biomass measured from the WT control (blue), WT cortisol (yellow), GRKO (purple), and MRKO (pink) liver at 1 h post-injection. **(E)** Whisker box plot showing the rate of endogenous glycolytic intermediate fructose-1,6-bisphosphate (F-1,6-BP) produced per unit of D-glucose (DG), expressed as the ratio of F-1,6-BP to DG calculated by dividing the relative abundances of F-1,6-BP by D-glucose. **(F)** Whisker box plot showing the rate of endogenous glycolytic product pyruvate produced per unit of D-glucose, expressed as the ratio of pyruvate to D-glucose calculated by dividing the relative abundances of pyruvate by D-glucose. Bars with a different letter indicate significant difference (one-way ANOVA, *p* < 0.05; *N* = 6). WT, wild-type control; WT+C, cortisol-treated wild type; GRKO, glucocorticoid receptor knockout (*nr3c1^−/−^*); MRKO+C, cortisol-treated mineralocorticoid receptor knockout (*nr3c2^−/−^*).

The parent global metabolites identified in the brain were subjected to dimension reduction PLS-DA. Although components 1 and 2 demonstrated 44.8% and 18.7% of the variability between the groups, respectively, the confidentiality Q2 score did not pass the 0.5 threshold (**Figure 9A**). Hence, all the identified 43 metabolites were subjected to a one-way ANOVA (Tukey’s HSD *post hoc*) and pathway analysis using the zebrafish-specific KEGG pathway. There were 32 metabolites that were significantly different and are shown as a heatmap depicting the relative abundance between the groups (**Figure 9B**). The abundances in the GRKO were higher for most of the listed metabolites and were evident by the contrast in the heatmap (**Figure 9B**). Pathway enrichment analysis was first performed between the WT to establish the effect of cortisol and then both GRKO and MRKO were compared separately with the cortisol-treated WT (**Table 2**). The 10 most affected pathways are shown in **Table 2**, and cortisol significantly affected 4 pathways, including glycolysis, tyrosine metabolism, phenylalanine, tyrosine, and tryptophan biosynthesis, and histidine metabolism. When GRKO and MRKO were compared with the cortisol-treated WT separately, almost all the 10 listed pathways were significantly different in GRKO, while only the TCA cycle pathway was significantly altered in MRKO compared to the cortisol-treated WT (**Table 2**).

**Table 2:** List of metabolic pathways in the brain significantly different between the treatments/genotypes when separately analyzed against cortisol-treated wild type.

Pathway	Match	Impact	WT vs. WT+C	WT+C vs. GRKO	WT+C vs. MRKO+C
			FDR	FDR	FDR
Glycolysis	4/26	0.17744	0.033729	5.4929E-4	0.2565
Phenylalanine, tyrosine, and tryptophan biosynthesis	1/4	0.5	0.0081331	0.074474	0.43642
Tyrosine metabolism	3/42	0.16435	0.025	9.3257E-4	0.35659
Histidine metabolism	2/17	0.22449	0.025	9.1296E-4	0.07784
Citrate cycle (TCA cycle)	6/20	0.30194	0.17626	9.1296E-4	0.027318
Alanine, aspartate, and glutamate metabolism	9/27	0.33989	0.17626	9.1296E-4	0.18106
Glutathione metabolism	5/28	0.30969	0.30452	5.4929E-4	0.5523
Valine, leucine, and isoleucine biosynthesis	2/40	0	0.36844	0.04997	0.2565
Arginine biosynthesis	6/14	0.2538	0.38512	0.0016996	0.673
Glycine, serine, and threonine metabolism	5/33	0.33036	0.5166	0.0096237	0.3924

The “Match” column shows the number of metabolites identified in respect to the total number of metabolites involved in each pathway. The “Impact” column shows the numerical representation of matched metabolites’ importance and centrality of a specific metabolic pathway, normalized to the sum of all the metabolites within the pathway. FDR, false discovery rate; WT, wild-type control; WT+C, cortisol-treated wild type; GRKO, glucocorticoid receptor knockout (*nr3c1^−/−^*); MRKO+C, cortisol-treated mineralocorticoid receptor knockout (*nr3c2^−/−^*).

Among the differentially affected pathways, tyrosine enrichment was identified in two pathways. Hence, the relative abundance of tyrosine was further investigated. The tyrosine pool size was 7-fold higher in the cortisol-treated WT brain compared to the control (*p* < 0.0001) and 2.5-fold greater than MRKO (*p* = 0.02; **Figure 9C**). The GRKO brain showed a 4-fold higher relative abundance of tyrosine from the WT control (*p* = 0.0008), but not from the other groups (**Figure 9C**). The enrichment of histidine metabolism pathway (2/17) consisted of Gln/Glu and histidine together having an impact of 0.225 (**Table 2**). The histidine pool size was 1.5-fold lower in the cortisol-treated WT compared to the WT control (*p* = 0.038; **Figure 9D**). GRKO had a ~2-fold higher relative abundance of histidine than the control and MRKO, and 3-fold higher than the cortisol-treated WT brain (*p* = 0.002, 0.003, and 0.0001, respectively; **Figure 9D**).

The glycolytic pathway was significantly affected by cortisol treatment in the WT, with an impact score of 0.178 and 4 identified metabolites (**Table 2**). To further understand the treatment effects on the glycolytic pathways, ratios of metabolite to common precursors were calculated as described previously (25). The fructose-1,6-bisphosphate (FBP)-to-brain D-glucose ratio was at the highest in MRKO and was significantly higher than the WT control, GRKO, and the cortisol-treated WT groups (*p* < 0.0001, 0.007, and 0.02 respectively; **Figure 9E**). The FBP-to-D-glucose ratio in the cortisol-treated WT was higher than that in the WT control (*p* = 0.046), but lower than that in MRKO (*p* = 0.016; **Figure 9E**). The pyruvate-to-D-glucose ratio was at the lowest in MRKO and was not significantly different from the control (*p* = 0.9; **Figure 9F**). The cortisol-treated WT brain showed a significantly higher pyruvate-to-D-glucose ratio compared to the WT control, GRKO, and MRKO (*p* = 0.0001, 0.02, and < 0.0001, respectively; **Figure 9F**). GRKO had a higher pyruvate-to-D-glucose ratio compared to only the MRKO brains (*p* = 0.03), and lower than the cortisol-treated WT (*p* = 0.02), but not different from the WT controls (*p* = 0.09; **Figure 9F**).

## 4 Discussion

**Figure 10:**
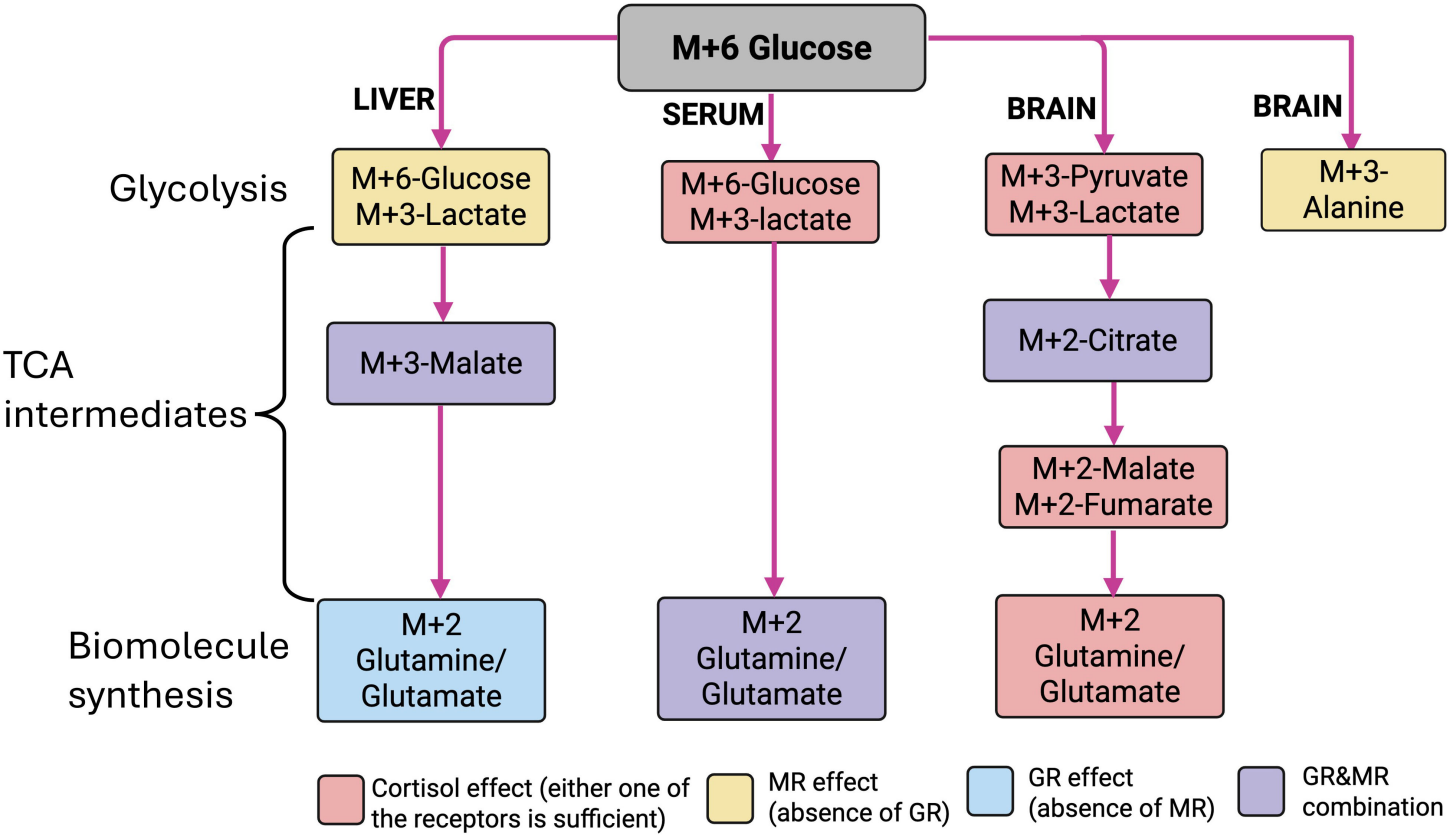
Schematic representation of the role of GR and MR in the tissue-specific generation of labeled intermediates from ^13^C-glucose. Fate of M + 6 glucose (gray rectangle) in liver, serum, and brain, showing the different labeled intermediate observed within glycolysis, TCA, and biomolecule synthesis. The pink rectangle represents cortisol effect (no specific receptor response); the yellow rectangle represents MR activation or the absence of GR; the blue rectangle represents GR activation or the absence of MR; the purple rectangle represents the presence (activation) of GR and MR. Image created in BioRender. Vijayan, M. (2025) https://BioRender.com/gu8y4ur.

Our results demonstrate that GR and MR signaling have distinct as well as complementary roles in bringing about the chronic cortisol-mediated regulation of glucose metabolism in zebrafish. Glucose is a preferred fuel to cope with the enhanced energy demand associated with stress, and cortisol modulates their production and utilization in fish (3, 4). A key finding from this study is that apart from the use of glucose as a metabolic fuel, it may also play an important role in the generation of biosynthetic molecules, including Gln/Glu in response to chronic cortisol stimulation. The abundance of Gln/Glu generation from glucose seen in the brain due to cortisol stimulation leads to the proposal that this metabolite may be a critical player in stress-related brain function, including behavioral changes (6, 7). To this end, glutamate levels increased in zebrafish brain in response to chronic environmental stress, and glutamine supplementation has been shown to mitigate stress and improve growth in aquaculture (44, 45). Overall, the intermediary metabolites generated from glucose in the brain and liver indicates a key role for both the GR and MR signaling in driving the cortisol-mediated tissue-specific metabolic adjustments associated with chronic stress in fish (**Figure 10**).

### 4.1 Cortisol-mediated metabolite regulation in circulation

Stress and cortisol increase the mobilization of glucose in the circulation to fuel the increased metabolic demands (4, 8). The rapid increase in circulating glucose is mediated by epinephrine stimulation of glycogenolysis, whereas the sustained elevation of this metabolite post-stressor is associated with cortisol-mediated gluconeogenesis (4, 12). The higher distribution of labeled lactate and Gln/Glu in the circulation in response to chronic cortisol stimulation points to a rapid utilization of glucose by target tissues for energy production and Gln/Glu synthesis in fish. The changes observed in these labeled serum metabolites in zebrafish lacking GR and MR underscore a key role for the corticosteroid receptors in regulating the energy substrate mobilization in response to chronic cortisol stimulation in fish.

Most studies on stress and cortisol point to a key role for GR in mediating the metabolic adaptations essential for coping with stress in fish (16, 46, 47). However, recently, we showed that MR also has an important role in modulating the cortisol-mediated tissue metabolic adjustments in zebrafish (6, 7). This was further supported by the observation that glucose uptake was higher in the skeletal muscle of fish lacking GR, which has a functional MR (7), indicating a key role for MR in tissue glucose metabolism (6). From our tracer results, the highest distribution of M + 6 glucose in the circulation was in fish lacking GR (**Figure 2B**), suggesting that this receptor may play an important role in the target tissue glucose uptake from circulation during stress in fish. However, whether this is mediated by a lack of GR and/or the activation of MR in specific tissues, including muscle, remains to be elucidated. Also, given that fish lacking GR are inherently hypercortisolemic, it remains to be seen whether the long-term developmental exposure to elevated cortisol in this genotype may have other metabolic consequences that are independent of GR activation.

The abundance of energy substrates generated from labeled glucose highlights a key role for cortisol not only in mobilizing the stored nutrients to produce glucose (4), but also in the efficient utilization of glucose for producing other energy substrates, including lactate and Gln/Glu (33, 48, 49). The production of these metabolites from glucose may be a key role for cortisol, as lactate and Gln/Glu are preferentially utilized for oxidation by the TCA cycle in several peripheral tissues, including gut, immune cells, and kidneys during both homeostasis and stress (48, 50–52). Consequently, chronic cortisol stimulation may enhance glucose turnover (4), and our results suggest that this glucose utilization may favor aerobic metabolism to cope with the increased energy demand associated with stress (52, 53).

In addition to their role as substrates for energy, lactate and Gln/Glu may also play a role in cell signaling and as cellular defense to cope with stress (51, 54). This is supported by selective increase in the labeled lactate and Gln/Glu by cortisol treatment both centrally and peripherally in the present study. For instance, we observed trace levels of M + 2 lactate (~1%) in the circulation within 1 h post-injection of the stable isotope (**Figures 2C**, **4B**). This rapid but low-abundance distribution suggests preferential lactate production through pathways involving CO_2_ recycling, such as anaplerotic fixation into oxaloacetate (32, 55). Lactate, a preferred TCA substrate in most peripheral tissues in both fish and mammals (48, 56), can also act as a signaling molecule by binding to hydrocarboxylic acid receptor 1 (HCAR1) (54, 56). This metabolite has also been shown to bring about epigenetic modification by lactylation, thereby regulating gene expression (54), including inhibition of fat breakdown and fatty acid oxidation (53). Increased lactate in the circulation has also been shown to reduce the rate of glycolysis (53), thereby modulating energy substrate utilization. Consequently, the breakdown of glucose to lactate may not only serve as a key energy substrate for ATP production, but also be involved in stress signaling, but the exact mechanism remains to be determined in fish.

Glutamine is a non-essential amino acid but shown to be important in reducing stress-related impacts in fish (44, 57). It is a multifaceted amino acid that can be converted to glutamate by glutaminase enzyme (52), and enters the TCA cycle via α-KG (24, 33). In addition to its role as an intermediate of energy production, this metabolite is also an important substrate for nucleotide synthesis, glutathione synthesis, pH homeostasis, and NADPH (nicotinamide adenine dinucleotide phosphate) generation (52). The glutamine–glutamate metabolic pathway plays a vital role in coping with oxidative stress (45). However, lactate and Gln/Glu levels in the circulation are tightly regulated (52, 53), and this is seen in the present study. Chronic cortisol stimulation did not affect the endogenous metabolite pool sizes, including lactate and Gln/Glu in the circulation. However, in the absence of GR and/or MR activation, the pool size of lactate and Gln/Glu was significantly higher, suggesting the importance of these receptors in the tight regulation of circulating metabolites during stress in fish.

### 4.2 Cortisol-mediated liver metabolite regulation

Liver is a major organ for metabolism, including glucose regulation, to support the increased energy demand associated with stress in fish (3, 4). Liver is also a major organ for glucose production, and the ~2.5% distribution of M + 3-glucose indicates the contribution of substrates derived from glucose metabolism for glucose recycling in fish. Although lactate (product of glucose breakdown) has been used as a substrate for gluconeogenesis in fish, amino acids are the more favored gluconeogenic substrates, especially in response to cortisol stimulation in fish (4). The ~1% M + 3 glucose in circulation points to the contribution of lactate as a substrate for the glucose pool (19, 48). The liver endogenous lactate pool size was higher with cortisol treatment, which could be from the breakdown of stored glycogen within the liver (58). While we did not measure liver glycogen content, a recent study showed that chronic cortisol treatment reduced muscle glycogen content in zebrafish (10), suggesting that this stress steroid enhances the glycolytic capacity leading to the increase in tissue lactate levels observed in fish (59). Interestingly, in the absence of either GR or MR, the endogenous liver lactate pool size was even higher, pointing to a role for the corticosteroid receptor activation in regulating this metabolite levels in response to chronic cortisol stimulation. We previously showed that a lack of GR did not affect muscle glycogen content (7), whereas the lack of MR reduced glycogen content in the zebrafish muscle (6). These studies suggest that both these receptors may be involved in glycolysis and lactate regulation observed in the present study. Our liver metabolite profile suggests that the elevated lactate pool size in the absence of GR could be from both increased glycolysis (lactate/glucose ratio; **Figure 3D**) and reduced TCA consumption (malate/lactate ratio; **Figure 5E**), whereas in the absence of MR, the lactate abundance is primarily from reduced TCA consumption (**Figure 3F**). Also, the circulating M + 3 lactate may arise from the skeletal muscle, as it is the principal peripheral tissue source for circulating lactate (58–60). The exact mechanism on how GR and MR together regulate the substrate utilization remains to be elucidated, but we propose that this may involve receptor interactions, including heterodimerization of the receptors.

The origin of circulating labeled Gln/Glu may be from the liver, as the WT-cortisol liver also showed a higher M + 2 Gln/Glu distribution (**Figure 4D**). Liver can store excess substrates from the circulation as nutrient reserves, while also breaking down stored energy depots during an energy deficit (61, 62). Liver glutamine biosynthesis has also been shown to increase with elevated cortisol and with acute stress in fish (63, 64). Therefore, it is possible that the elevated circulating labeled Gln/Glu observed in the cortisol group could be of hepatic origin. Interestingly, in the cortisol group, despite an enhanced M + 2 Gln/Glu cataplerosis (33), they were able to sustain the TCA activity to meet the higher energy demand, as evident from the higher M + 3 malate distribution, which possibly is replenished through pyruvate recycling via oxaloacetate (**Figure 2A**; 24, 33). This was further supported by a higher endogenous fumarate-to-Gln/Glu ratio in the cortisol-treated liver (**Figure 6D**), suggesting a possible increase in anaplerosis (37, 52). However, this was seen only in the presence of both GR and MR (WT fish treated with cortisol) because in the absence of GR, an increase in either labeled Gln/Glu or malate was not observed. Also, in the absence of MR, only an increase in labeled Gln/Glu but not malate was observed, indicating a key role for the activation of both receptors in mediating the metabolic flux through the TCA cycle.

### 4.3 Cortisol-mediated metabolite regulation in the brain

Brain exhibits the highest energy demand, accounting for nearly ~15% to 20% of the oxygen consumed despite constituting only ~2% of the body mass in vertebrates (39, 65). As in mammals, teleost brain also depends heavily on the circulating glucose to fuel the energy needs, and this is evident from the high glycolytic capacity of this organ (66). Our results indicate a high glucose utilization capacity, as there was very little ^13^C-glucose recovery in the brain at 1 h post-injection of labeled glucose relative to that seen in the liver. Also, the labeled intermediates generated from glucose, including pyruvate, lactate, and alanine, support an enhanced glycolytic capacity (66–68), and these changes in the brain were clearly modulated by the activation of corticosteroid receptors. The lack of GR further enhanced the glucose flux in generating these glycolytic intermediates’ distribution, indicating a role for this receptor in modulating brain glycolysis (**Figures 7A–D**).

The generation of citrate from glucose, indicative of flux towards the TCA cycle (69) in the brain, was not affected by chronic cortisol stimulation; however, the labeled citrate pool was lower in the absence of GR or MR, suggesting a role for these receptors in regulating brain oxidative metabolism in response to chronic cortisol stimulation. The subsequent labeled TCA intermediates, including α-KG, succinate, fumarate, and malate, were not heavily affected by chronic cortisol treatment or the genotypes in the present study. One possible explanation may be that the TCA is a closed loop; thus, several intermediates often exit the cycle for biomolecule synthesis via cataplerosis and are also replaced at different levels to sustain the metabolic pathway via anaplerosis (24, 33). In the present study, the lack of changes in the subsequent M + 2 TCA intermediates despite a reduced labeled citrate in the absence of GR or MR could be from the Gln/Glu anaplerosis (33). The possible source of M + 2 Gln/Glu for anaplerosis in the GRKO and MRKO brain could be from a higher uptake from the circulation. This could have also resulted in a reduced M + 2 Gln/Glu in the circulation in the absence of GR or MR, but this remains to be investigated.

In the present study, in addition to enhanced glycolytic and oxidative capacity of the brain, we also observed a relative increase in the phosphorylated pathway activity from glycolysis as evidenced by the presence of M + 2- and M+1-labeled O-phospho-L-serine (**Figure 7D**). Phosphoserine is generated from the glycolytic intermediate 3-phosphoglycerate in the process of *de novo* L-serine biosynthesis (41). The irreversible conversion of phosphoserine to L-serine is dictated by the demand and abundance of L-serine levels in the human brain (41). Here, we observed a reduced endogenous phosphoserine in the cortisol-treated WT, suggesting an increased L-serine biosynthesis and consumption. The MRKO group exhibited a trend similar to that observed in cortisol-treated WT, although this difference was not statistically significant from the WT controls. In contrast, the GRKO group showed no changes when compared to the WT controls. These results suggest that both GR and MR may be involved in regulating *de novo* L-serine synthesis and/or consumption in the brain. L-serine is an essential amino acid to neurons, which is exclusively synthesized by the astrocytes via glycolysis (70). The significance of phosphorylated pathway and L-serine synthesis from glycolysis has been extensively studied as a critical player in neurotransmission and neuroprotective functions in humans (71, 72). To the best of our knowledge, there are no studies in fish showing a similar significance of L-serine biosynthesis, but the phosphorylated pathway is extensively conserved across the eukaryotes (71). The results from the present study showing the differences in endogenous phosphoserine abundance and distribution in the absence of GR and/MR activation will prompt further investigation on the phosphorylated pathway in fish brain and its significance in stress coping.

Glutamine, the most important precursor of glutamate, is a critical neurotransmitter and the most abundant amino acid in the brain (42, 43). In any given time, an approximate concentration of 10 mmol of glutamate is observed per kilogram of human brain (42). The significance of Gln/Glu can be reflected by the observation of all possible six isotopologues of Gln/Glu in the brain tissue at 1 h post-injection (**Figure 8D**). The M + 2 Gln/Glu isotopomere was generated directly from the ^13^U-glucose by the entry of pyruvate via acetyl CoA (**Figure 2A**) and was similar across GRKO, MRKO, and the cortisol-treated WT, suggesting that one of the CRs is sufficient for the cataplerotic exit of α-KG in the brain. However, the endogenous Gln/Glu in GRKO is higher than that in MRKO and cortisol-treated WT, but lower than that in the WT control. In mammals, GR has been shown to increase glutamate release for the neuronal consumption (73), whereas in zebrafish larvae, GC deficiency caused glutamine accumulation due to reduced glutaminolysis and the associated glutaminase enzyme expression (74). These findings suggest an important role for cortisol-GR in the Gln/Glu turnover, as this was not observed in fish lacking MR, which showed a metabolite distribution similar to that of the WT-cortisol fish. Similarly, we observed M + 3 labeling of malate and fumarate, which could be from pyruvate recycling anaplerosis via oxaloacetate (**Figure 2A**; 24, 33). These anaplerotic replenishments were not affected between the genotypes. Both the processes of anaplerosis in replenishing the lost TCA intermediates and the Gln/Glu cataplerosis in sustaining the neurotransmitter biosynthesis (43) were maintained in the zebrafish brain by the activation of either of the CRs.

An interesting observation from this study is the selective upregulation of tyrosine and a reduction in histidine pool size in response to chronic cortisol stimulation. These changes were modulated distinctly by MR and GR; absence of MR but not GR led to a decrease in tyrosine pool size, while the absence of GR increased histidine pool size. The physiological significance of these amino acids is their importance as precursors for neurotransmitter biosynthesis (75, 76). For instance, histidine is converted to histamine, while tyrosine is the precursor of catecholamine biosynthesis (75, 76). Zebrafish studies highlighted that an increase in histidine supplementation showed a linear increase in brain histamine levels (76), while tyrosine supplementation increased catecholamine levels in the fish brain during chronic stress (77). Furthermore, histaminergic and catecholaminergic neurons are found in close proximity within the vertebrate hypothalamus and telencephalon of the zebrafish brain (76). The reciprocal control of brain histamine and tyrosine levels by elevated cortisol may be critical in the distinct activation of stress appraisal, initiation, and termination pathways, and warrants further study. While our study is the first to address the contribution of GR and MR signaling in shaping the metabolic fate of glucose during chronic stress stimulation, there were a few limitations. For instance, we did not test glucose flux in the muscle, a key target tissue for cortisol action during stress (4, 6, 7). Also, we only used male fish in this study, which precluded us from inferring whether the observed effects were a generalized response or sex-specific.

In summary, the present study highlights the tissue-specific glucose regulation by GR and/or MR activation in response to chronic cortisol stimulation for stress coping. The glucose metabolism may involve not only ATP production but also the generation of biomolecules, including neurotransmitters, to facilitate stress adaptation. For instance, consider the glucose-generated Gln/Glu observed in the circulation, the liver, and the brain. Liver Gln/Glu biosynthesis needs the activation of GR (but not the MR), while in the brain, either one of the CR activation is sufficient for the Gln/Glu biosynthesis. However, both receptors are needed for the elevated circulating Gln/Glu generated from glucose, pointing to a tissue-specific control of metabolite regulation by CRs in response to chronic cortisol stimulation (**Figure 10**). Similar tissue specificity is observed with the sustenance of TCA capacity. The liver anaplerotic replenishment of the TCA intermediate (M + 3 malate) was observed only in the presence of both receptors’ activation, whereas in the brain, anaplerotic replenishment only required either one of the CRs (**Figure 10**). While the overall metabolic pathways may not provide a consistent picture, the specific pathway changes seen with isotope tracing are clear and significant, highlighting the advantages and reliability of the stable isotope approach for revealing the metabolic flux in tissues. Altogether, our findings provide novel insights into the distinct and complementary role of CRs in modulating glucose metabolism in response to chronic cortisol stimulation. While the role of cortisol in promoting substrate availability during stress and increases in metabolic rate is well known (78, 79), this study highlights a key role for both the corticosteroid receptors in fine-tuning the metabolic processes both centrally and peripherally. Our results, along with those of other studies that have used GR and MR knockout models in zebrafish, suggest the interaction between these two receptors as key in shaping the physiological and behavioral effects with cortisol in fish (6, 7, 15, 17, 18, 46, 80–83). In conclusion, the interaction of GR and MR is essential to sustain circulating substrates, including lactate and Gln/Glu, to allow metabolic adjustments to chronic stress in fish. At the tissue level, MR and GR work together to enhance metabolic capacity without impairing the production of key signaling molecules such as Gln/Glu (**Figure 10**). These signaling molecules, in addition to being an energy substrate, may exert extra-metabolic functions, including neurotransmission and cellular stress protection during chronic stress.

## Data Availability

The raw data supporting the conclusions of this article will be made available by the authors, without undue reservation.

## Supplementary material

Supplementary Figure 1 Stacked bar graph showing the mass isotopologue distribution (MID) of alanine measured in the liver at 1-h post injection of labeled glucose. Different color represents different isotopologue, expressed as percentage in respect to the total pool size (labeled and unlabeled) of the respective metabolite. The legend for each color corresponding to each isotopologue in the stacked bar is shown on the top right corner of the graph and identified as M+(n), where (n) indicates the number of ^13^C incorporated into each metabolite isotopologue. No significant differences were observed (two-way Anova, P<0.05; N = 5). WT, wildtype control; WT+C, cortisol-treated wildtype; GRKO, glucocorticoid receptor knockout (*nr3c1^-/-^*); MRKO+C – cortisol-treated mineralocorticoid receptor knockout (*nr3c2^-/-^*).

Supplementary Figure 2 Stacked bar graph showing the mass isotopologue distribution (MID) of α-ketoglutarate (α-KG) **(A)** and succinate **(B)** measured in the brain at 1-h post injection of labeled glucose. Different color represents different isotopologue, expressed as percentage in respect to the total pool size (labeled and unlabeled) of the respective metabolite. The legend for each color corresponding to each isotopologue in the stacked bar is shown on the top right corner of the graph and identified as M+(n), where (n) indicates the number of ^13^C incorporated into each metabolite isotopologue. No significant differences were observed (two-way Anova, P<0.05; N = 5). WT, wildtype control; WT+C, cortisol-treated wildtype; GRKO, glucocorticoid receptor knockout (*nr3c1^-/-^*); MRKO+C – cortisol-treated mineralocorticoid receptor knockout (*nr3c2^-/-^*).
