# Supplementary figures and images for: Stable-isotope tracing reveals the role of corticosteroid receptors in driving cortisol-mediated central and peripheral glucose regulation in zebrafish
Source: Front Endocrinol (Lausanne). 2025 Oct 14;16:1670637. doi: 10.3389/fendo.2025.1670637 (PMC12558738; doi:10.3389/fendo.2025.1670637)

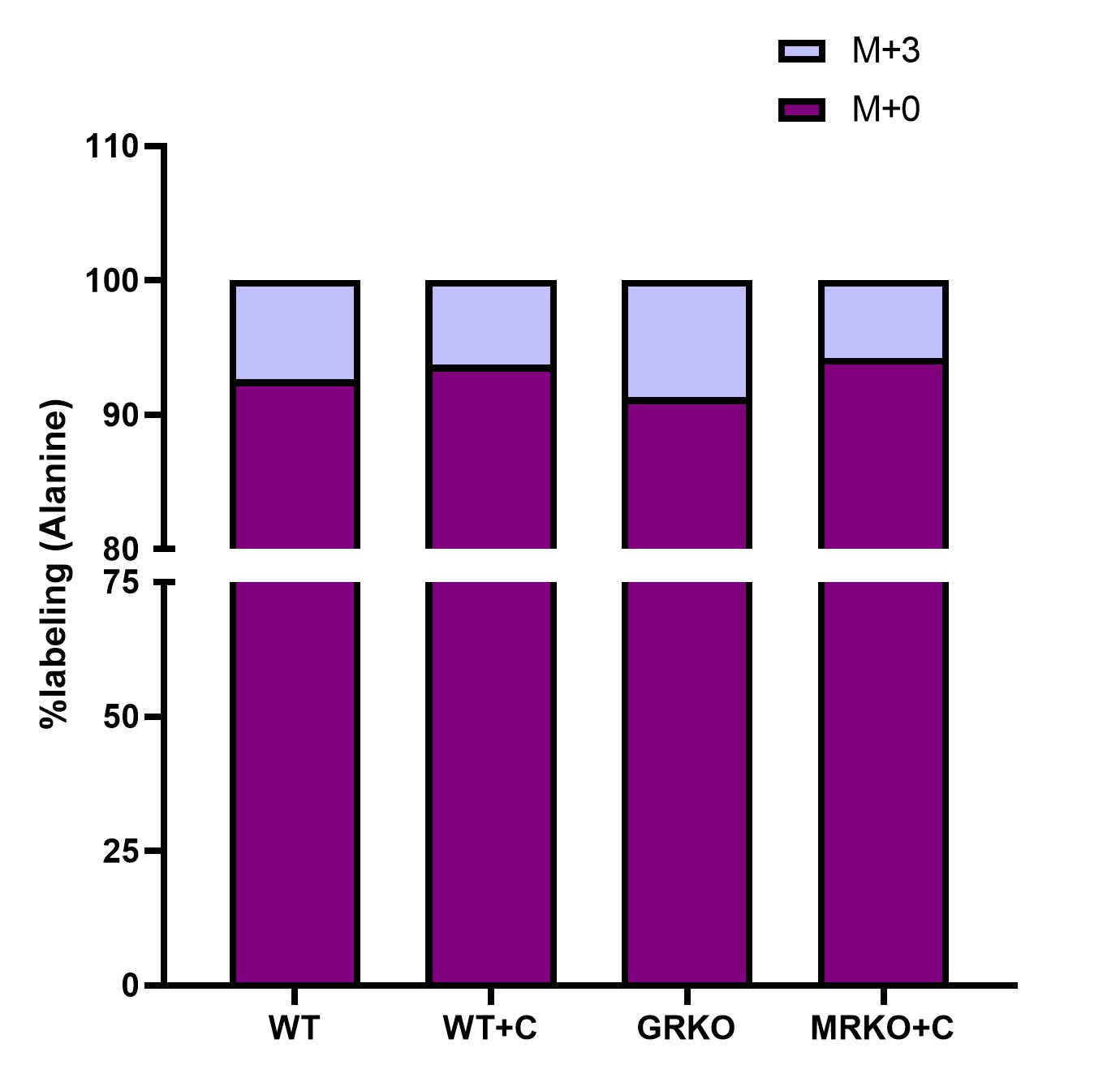

Supplement: Supplementary Figure 1 — Stacked bar graph showing the mass isotopologue distribution (MID) of alanine measured in the liver at 1-h post injection of labeled glucose. Different color represents different isotopologue, expressed as percentage in respect to the total pool size (labeled and unlabeled) of the respective metabolite. The legend for each color corresponding to each isotopologue in the stacked bar is shown on the top right corner of the graph and identified as M+(n), where (n) indicates the number of 13C incorporated into each metabolite isotopologue. No significant differences were observed (two-way Anova, P<0.05; N = 5). WT, wildtype control; WT+C, cortisol-treated wildtype; GRKO, glucocorticoid receptor knockout (nr3c1-/- ); MRKO+C – cortisol-treated mineralocorticoid receptor knockout (nr3c2-/- ). [file Image1.jpeg]

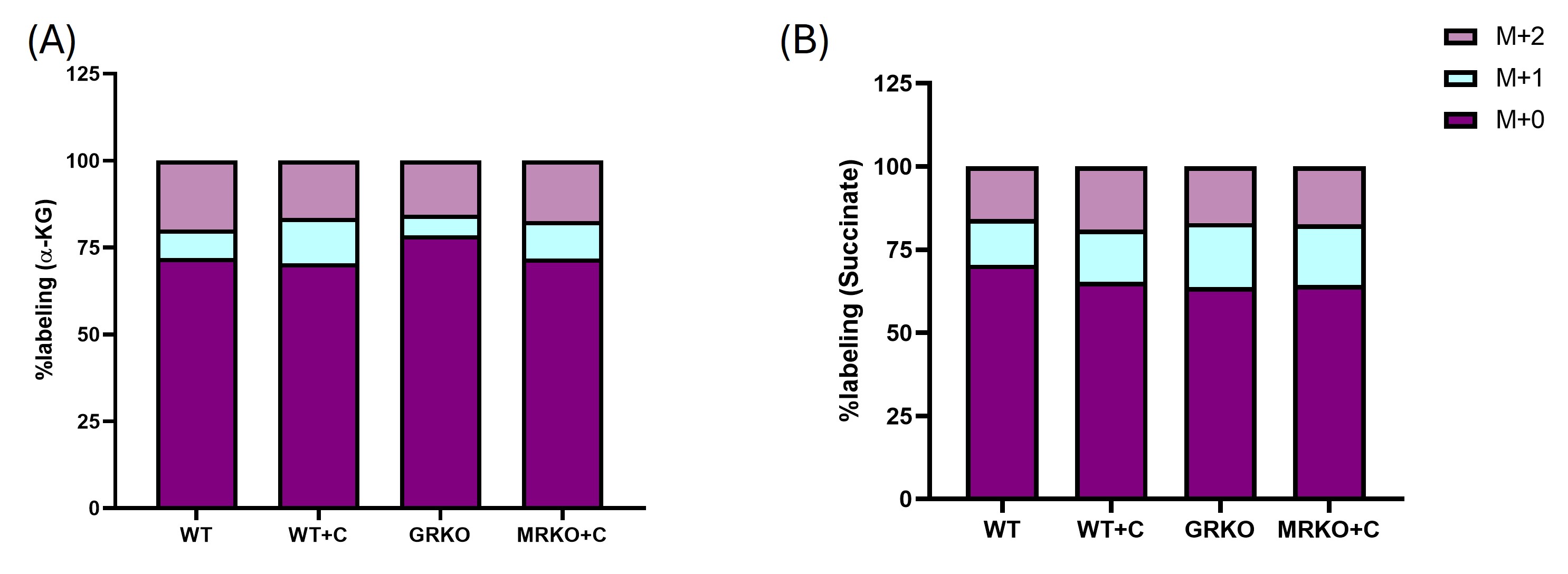

Supplement: Supplementary Figure 2 — Stacked bar graph showing the mass isotopologue distribution (MID) of α-ketoglutarate (α-KG) (A) and succinate (B) measured in the brain at 1-h post injection of labeled glucose. Different color represents different isotopologue, expressed as percentage in respect to the total pool size (labeled and unlabeled) of the respective metabolite. The legend for each color corresponding to each isotopologue in the stacked bar is shown on the top right corner of the graph and identified as M+(n), where (n) indicates the number of 13C incorporated into each metabolite isotopologue. No significant differences were observed (two-way Anova, P<0.05; N = 5). WT, wildtype control; WT+C, cortisol-treated wildtype; GRKO, glucocorticoid receptor knockout (nr3c1-/- ); MRKO+C – cortisol-treated mineralocorticoid receptor knockout (nr3c2-/- ). [file Image2.jpeg]
